# Optimal designs for discrete-time survival models with competing risks

**DOI:** 10.1007/s10985-026-09695-0

**Published:** 2026-02-28

**Authors:** XiaoDong Zhou, YunJuan Wang, RongXian Yue, Weng Kee Wong

**Affiliations:** 1 School of Statistics and Data Science, Shanghai University of International Business and Economics, Shanghai, 201620 China; 2 School of Statistics and Mathematics, Shanghai Lixin University of Accounting and Finance, Shanghai, 201620 China; 3 School of Arts and Sciences, Fuyao University of Science and Technology, Fuzhou, 350109 China; 4 Department of Biostatistics, University of California, Los Angeles, Los Angeles, CA 90095-1772 USA

**Keywords:** Competing risks, Discrete-time survival model, Longitudinal study, Parametric competing risks model, Time-varying treatment effects., MSC 62K05

## Abstract

Current methodological research on randomized controlled trial design has predominantly focused on studies with a single primary endpoint. However, many trials in practice involve multiple competing target events. The optimal designs for survival trials with competing target events have not been systematically addressed in the statistical literature. This paper fills this significant gap by developing design methodologies for randomized discrete-time-to-event trials with competing endpoints. We derive the Fisher information matrix for the general discrete-time survival model (DTSM) by transforming the original discrete-time survival data into proper multinomial responses. By introducing a cost-based generalized $D_A$-optimal design criterion, we identify various types of optimal designs for estimating the treatment effects. Under the assumption of a parametric competing risks model for the underlying survival process, we demonstrate that the optimal treatment allocation scheme is critically influenced by the parameter values within this model. Our methodology is applied to the redesign of the SANAD trial, which examines withdrawal times from anti-epileptic drugs, thereby highlighting the advantages of our optimal design strategies. A key finding is that assigning subjects equally to the different groups in a two-arm DTSM trial with competing risks is generally a favorable choice, unless the hazard rates over the duration of the trial in both groups are low.

**Supplementary Information:**

The online version contains supplementary material available at 10.1007/s10985-026-09695-0.

## Introduction

Longitudinal clinical trials employ randomized allocation of participants into experimental and control arms to evaluate treatment effects over extended temporal intervals, with the former receiving the investigational therapy and the latter administered either standard care or placebo. The optimization of such trial designs has been explored by many researchers, considering different application scenarios based on various statistical models (Mentré et al. 1997; Debusho and Haines 2008; Ji and Müller 2017; Galbraith et al. 2017; Lee et al. 2019; Yi et al. 2022; Hartley et al. 2024).

In the aforementioned studies, researchers focused on endpoints that were assumed to be precisely measured or observed at predetermined time points, quantifiable in units such as minutes, hours, days, weeks, months, or even years. However, this is not always the case in real-world scenarios. In many instances, survival data is measured discretely, meaning it is grouped into specific time intervals rather than being continuously measured. For example, in clinical trials that address time to pregnancy, the observation period is often defined by the number of menstrual cycles, representing a discrete time interval (Fehring et al. 2013). Data of this type, known as discrete-time or grouped-time survival data, can be readily analyzed using discrete-time survival models. These models formulate the problem within a generalized linear model framework (e.g., logistic regression) or leverage modern machine learning techniques (e.g., tree-based methods) (Tutz and Schmid 2016; Suresh et al. 2022), regardless of whether the underlying survival process is continuous or discrete.

Recently, optimal designs for longitudinal studies with discrete-time survival endpoints have been studied by various authors. Jóźwiak and Moerbeek (2012, 2013) constructed cost-effective designs for DTSMs and identified optimal treatment allocation schemes and study duration. Additionally, Safarkhani and Moerbeek (2014) investigated the influence of a covariate can have on optimal designs in DTSMs. Moerbeek and Wong (2015) found optimal treatment allocations for longitudinal trials to compare multiple treatment groups with a placebo group. Further, Safarkhani and Moerbeek (2015) addressed design challenges in trials where treatment effects vary over time, and they subsequently found *D*-optimal designs for estimating parameters in longitudinal DTSMs with a single quantitative predictor variable (Safarkhani and Moerbeek 2016). Finally, Zhou et al. (2021) found optimal designs for DTSMs in the presence of heterogeneity among subjects.

Previous research has predominantly focused on basic survival models for a single event of interest. However, in practice, multiple competing events are often of concurrent interest. For instance, in studies of Hodgkin’s disease patients treated with radiation or combined modalities, key long-term outcomes include time to first relapse, occurrence of a second malignancy, and overall survival (Pintilie 2006). Statistical frameworks for such data are known as competing risks models (Fahrmeir and Wagenpfeil 1996; Austin et al. 2016), and methodology development in this area remains highly active. Schmid and Berger (2021) provides a comprehensive overview of discrete-time survival analysis with competing risks. Recent methodological advances addressing various aspects of these models include: a sufficient discrete hazard approach for time-varying covariates (Wen and Chen 2020); methods for evaluating discrimination, calibration, and prediction error (Heyard et al. 2020); techniques for assessing the calibration of discrete time-to-event models (Berger and Schmid 2022); and a multivariate Bernoulli detector incorporating change-point modeling (van den Boom et al. 2024). Furthermore, Monterrubio-Gómez et al. (2024) offers a timely review that bridges traditional statistical and modern machine learning methods for competing risks, encompassing both continuous and discrete-time survival data.

Interest in addressing estimation and analysis issues often precedes the consideration of design issues. This trend holds true for DTSMs with competing risks, where attention to design issues is only just beginning to emerge. This focus on design is crucial because the accuracy of statistical inferences relies heavily on the design employed; an optimal design enables the most accurate inferences to be made at minimum cost. Therefore, in this paper, we develop techniques for finding optimal designs for DTSMs with competing risks and use the multinomial logit model to relate the risks to the explanatory variables (King and Weiss 2021).

To the best of our knowledge, this problem has not been holistically treated in the literature. Although earlier works such as Zocchi and Atkinson (1999) and Bu et al. (2020) employed multinomial logistic models in design settings, which can be viewed as a special case of single-interval DTSMs with competing risks, their scope was restricted to a single inspection time. Our work extends this line of inquiry to a more general and practical setting with multiple inspection intervals. Specifically, we address the optimal determination of the number of inspections, group allocation weights, and sample sizes under a fixed total cost.

Our methodological choice of a discrete-time modeling framework is motivated by both conceptual and practical considerations. Unlike continuous-time models that target instantaneous hazards, discrete-time models estimate interval-specific conditional event probabilities (Singer and Willett 2003). This formulation not only aligns with studies involving periodic follow-ups and tied event times but also facilitates the tractable derivation of optimal designs within a generalized linear model framework, similar to its utility in modern machine learning applications (Suresh et al. 2022; Schmid and Berger 2021). Within this paradigm, the multinomial logit model emerges as a natural representation for competing risks, as it directly captures the mutually exclusive event outcomes at each time interval.

The key contributions of this paper are as follows. We introduce the first unified design framework for randomized DTSMs with competing events, which is flexible enough to accommodate multi-arm trials and diverse study objectives. We also evaluate conventional uniform designs commonly used in two-arm competing risks studies. While these are shown to perform robustly in many cases, their efficiency declines in settings with low competition among risks. We believe the proposed approach offers practical and theoretical guidance for designing future studies with discrete-time survival endpoints subject to competing risks.

The paper is structured as follows. Section 2 describes the DTSM with competing risks and provides details for the Fisher information matrix (FIM) for the model. In Sect. 3, we discuss design concepts, cost functions and optimality criteria, and formulate the design problem that incorporates a cost function along with the $D_{\textbf{A}}$-optimal design. Section 4 presents simulation results, where we investigate two special models with two target events. In Sect. 5, we present a real-life case study demonstrating the effectiveness of our proposed design methods. Finally, We summarize our findings in Sect. 6, along with future interesting design work for DTSMs.

## Discrete-time survival models with competing risks

Suppose *J* is the total number of event types and $$R \in \{1, 2,\ldots , J\}$$ denotes the *J* distinct target events. Let *q* be the maximum number of time periods and the *q* underlying time periods are $$[a_0,a_1), [a_1,a_2),\ldots $$ and $$[a_{q-1},a_q)$$. For a given discrete time $$T \in \{1,2, \ldots , q\}$$, the cause-specific hazard function associated with cause or risk *r* at time *t* is given by:

1 $$\begin{aligned} \lambda _{r}(t | \textbf{x})=P(T=t, R=r | T \ge t, \textbf{x}), \end{aligned}$$

where $\textbf{x}$ represents a vector of time-independent covariates. The cause-specific hazard function represents the conditional probability that the event of type *r* occurs at time *t*, given that the patient has not experienced any event up to time $t-1$, while accounting for the covariates $\textbf{x}$. The overall hazard function describes the process, irrespective of the type of event, can be defined as the sum of the cause-specific hazard functions:

$$ \lambda (t | \textbf{x})=\sum _{r=1}^{J} \lambda _{r}(t | \textbf{x})=P(T=t | T \ge t, \textbf{x}). $$

The survival function and the unconditional probability function of an event occurring in time period *t* are then, respectively, given by

$$ S(t | \textbf{x})=P(T>t | \textbf{x})=\prod _{s=1}^t(1-\lambda (s | \textbf{x})) $$

and

$$ P(T=t | \textbf{x})=\lambda (t |\textbf{x}) S(t-1 |\textbf{x}). $$

The implication is that when an individual reaches the inspection interval $$[a_{t-1},a_t)$$, there are *J* possible outcomes: the occurrence of one of the *J* target events, or survival beyond the interval $$[a_{t-1},a_t)$$. The corresponding conditional response probabilities are

$$ \lambda _{1}(t | \textbf{x}), \ldots , \lambda _{J}(t | \textbf{x}), 1-\lambda (t | \textbf{x}), $$

where $1-\lambda (t | \textbf{x})$ represents the probability of survival beyond time *t*.

Furthermore, the discrete cumulative incidence function (CIF) for the *r*th target event, can be written as follows:

2 $$\begin{aligned} F_r(t|\textbf{x})=P(T\le t,R=r|\textbf{x})=\sum _{s=1}^t\lambda _r(s|\textbf{x})S(s-1|\textbf{x}). \end{aligned}$$

### Multinomial logit model

In discrete survival models, the responses are either the target events or survival. The discrete-time cause-specific hazard function $\lambda _{r}(t | \textbf{x})$ for target event *r* can be modeled using a multinomial logit model (Heyard et al. 2020; King and Weiss 2021), which is given by

3 $$\begin{aligned} \lambda _{r}(t | \textbf{x})=\frac{\exp \left( \gamma _{0 t r}+\textbf{f}_r^\mathrm{{T}}(\textbf{x}) {\boldsymbol{\gamma }}_{r}\right) }{1+\sum _{j=1}^{J} \exp \left( \gamma _{0 tj}+\textbf{f}_j^\mathrm{{T}}(\textbf{x}) {\boldsymbol{\gamma }}_{j}\right) }, r=1,\cdots ,J, t=1,\cdots ,q, \end{aligned}$$

where $\textbf{f}_j(\textbf{x})$ is a vector of known regression functions of $\textbf{x}$. The parameters $\gamma _{01r}, \dots , \gamma _{0 qr}$ represent the cause-specific baseline coefficients, and ${\boldsymbol{\gamma }}_r$ is the cause-specific vector of regression coefficients. We set the reference category be conditional survival, and its probability is

$$ \lambda _{J+1}(t|\textbf{x})=P(T>t | T \ge t, \textbf{x})=\frac{1}{1+\sum _{j=1}^{J} \exp \left( \gamma _{0 t j}+\textbf{f}_j^\mathrm{{T}}(\textbf{x}){\boldsymbol{\gamma }}_{j}\right) }. $$

For additional explanations regarding this model, please refer to Chapter 8 in Tutz and Schmid (2016).

### Cumulative logit model

In certain cases, causes or risks can be naturally ordered; for example, in terms of the severity of the event. For instance, in a trauma clinical study examining treatment outcomes for a medical condition, the target events $R \in \{1, 2, \ldots , J\}$ could represent different levels of treatment success, such as “vegetative state," “major disability," “minor disability," and “good recovery," with “death" denoted by $R = J + 1$ (Bu et al. 2020; Chuang-Stein and Agresti 1997). In such cases, the outcomes are ordered categorical responses and typically cumulative probabilities defined by

$$\pi _r(t|\textbf{x})=\sum _{j=1}^r\lambda _j(t|\textbf{x})=P(T=t,R\le r|T\ge t,\textbf{x}),r=1,\cdots ,J$$

are directly used in the model as follows:

4 $$\begin{aligned} \log \left( \frac{\pi _{r}(t | \textbf{x})}{1-\pi _{r}(t | \textbf{x})}\right) =\gamma _{0 t r}+\textbf{f}_r^\mathrm{{T}}(\textbf{x}){\boldsymbol{\gamma }},r=1,\cdots ,J, \end{aligned}$$

and the intercepts satisfy $\gamma _{0tr}\le \gamma _{0t,r+1}$ for all *r*. One advantage of this model, compared to the multinomial logit model, is that it is a more parsimonious model in that it requires only one vector of parameters ${\boldsymbol{\gamma }}$ instead of a separate vector of parameters ${\boldsymbol{\gamma }}_r$ for each category.

### Unified hazard formulation

The multinomial logit model (Sect. 2.1) and the cumulative logit model (Sect. 2.2), while employing different link functions, share a common structural foundation for the cause-specific hazards. We can synthesize these specific instances into a unified hazard formulation. The primary purpose of this generalization is to derive a single, compact expression for the FIM in Sect. 2.4, which will underpin our entire optimal design theory.

We present the unified formulation for the cause-specific hazard functions as:

$$ \lambda _{r}(t |\textbf{x})=h_{r}\left( \textbf{X}_{t} {\boldsymbol{\theta }}\right) , $$

where $$h_r$$ is a response function for responses in the interval $$[a_{t-1},a_t)$$, $$\textbf{X}_{t}$$ is a design matrix constructed from the covariate vector $$\textbf{x}$$, whose elements may be time-varying, and $${\boldsymbol{\theta }}$$ is a parameter vector which will be specified later.

For the multinomial logit model, let $\eta _r(t|\textbf{x})=\gamma _{0 t r}+\textbf{f}_r^\mathrm{{T}}(\textbf{x}) {\boldsymbol{\gamma }}_{r}, r=1,2,\ldots ,J$. To ensure identifiability, we define $\eta _{J+1}(t|\textbf{x})=0$. Then

$$ h_{r}\left( \textbf{X}_{t} {\boldsymbol{\theta }}\right) =\frac{\exp \left( \eta _{r}(t|\textbf{x})\right) }{1+\sum _{j=1}^{J} \exp \left( \eta _{j}(t|\textbf{x})\right) },r=1,\ldots ,J, $$

and the design matrix is

5 $$\begin{aligned} \textbf{X}_t=\left[ \begin{array}{cccccccccccccc} 0& \quad \ldots & \quad 1& \quad & \quad & \quad & \quad \ldots & \quad 0& \quad \textbf{f}_1^\mathrm{{T}}(\textbf{x})& \quad & \quad & \quad & \quad \\ 0& \quad \ldots & \quad & \quad 1& \quad & \quad & \quad \ldots & \quad 0& \quad & \quad \textbf{f}_2^\mathrm{{T}}(\textbf{x})& \quad & \quad & \quad \\ 0& \quad \ldots & \quad & \quad & \quad \ddots & \quad & \quad \ldots & \quad 0& \quad & \quad & \quad \ddots & \quad & \quad \\ 0& \quad \ldots & \quad & \quad & \quad & \quad 1& \quad \ldots & \quad 0& \quad & \quad & \quad & \quad \textbf{f}_J^\mathrm{{T}}(\textbf{x})& \quad \\ 0& \quad \ldots & \quad & \quad & \quad & \quad 0& \quad \ldots & \quad 0& \quad & \quad & \quad & \quad & \quad 1 \end{array} \right] , \end{aligned}$$

where a 1 in *r*th row of $\textbf{X}_t$ is at the $[(t-1)J+r]$th position. The vector of model parameters ${\boldsymbol{\theta }}$ is given by ${\boldsymbol{\theta }}^\mathrm{{T}}=\left( \gamma _{011}, \ldots , \gamma _{01 J}, \gamma _{021}, \ldots , \gamma _{0 q J}, \boldsymbol{\gamma }_{1}^\mathrm{{T}}, \ldots , \boldsymbol{\gamma }_{J}^\mathrm{{T}},0\right) .$

For the cumulative logit model, let $\eta _r(t|\textbf{x})=\gamma _{0 t r}+\textbf{f}_r^\mathrm{{T}}(\textbf{x}){\boldsymbol{\gamma }}$, and we have

$$\begin{aligned} h_{r}\left( \textbf{X}_{t} {\boldsymbol{\theta }}\right) =\textrm{logit}^{-1}\left( \eta _{r}(t|\textbf{x})\right) - \textrm{logit}^{-1}\left( \eta _{r-1}(t|\textbf{x})\right) , r=1,2,\ldots ,J, \end{aligned}$$

where $\textrm{logit}^{-1}(\eta )={e^\eta }/(1+e^\eta )$ and $\textrm{logit}^{-1}\left( \eta _{0}(t|\textbf{x})\right) =0$. In this case, the design matrix $\textbf{X}_t$ is defined by:

6 $$\begin{aligned} \textbf{X}_t=\left[ \begin{array}{cccccccccccccc} 0& \quad \ldots & \quad 1& \quad & \quad & \quad & \quad \ldots & \quad 0& \quad \textbf{f}_1^\mathrm{{T}}(\textbf{x})& \quad 0\\ 0& \quad \ldots & \quad & \quad 1& \quad & \quad & \quad \ldots & \quad 0& \quad \textbf{f}_2^\mathrm{{T}}(\textbf{x})& \quad 0\\ 0& \quad \ldots & \quad & \quad & \quad \ddots & \quad & \quad \ldots & \quad 0& \quad \vdots & \quad 0\\ 0& \quad \ldots & \quad & \quad & \quad & \quad 1& \quad \ldots & \quad 0& \quad \textbf{f}_J^\mathrm{{T}}(\textbf{x})& \quad 0\\ 0& \quad \ldots & \quad & \quad & \quad & \quad 0& \quad \ldots & \quad 0& \quad \textbf{0}^{\text {T}}& \quad 1 \end{array} \right] , \end{aligned}$$

and the corresponding parameter vector ***θ*** is ${\boldsymbol{\theta }}^\mathrm{{T}}$ $= \left( \gamma _{011}, \ldots , \gamma _{01 J}, \gamma _{021}, \ldots , \gamma _{0 q J}, \boldsymbol{\gamma },0\right) .$

Note that the vector of predictors $\textbf{x}$ in the risk functions (1) may include time-dependent covariates (See Model (18)). To accommodate this, the covariate vector in (5) or (6) can be generalized to $\textbf{x}_t$, explicitly allowing its values to change with the measured time interval *t*.

Let ${\boldsymbol{\lambda }}_{t}^\mathrm{{T}}=(\lambda _1(t|\textbf{x}),\ldots ,\lambda _J(t|\textbf{x}),\lambda _{J+1}(t|\textbf{x}))$. Following Zocchi and Atkinson (1999) and Bu et al. (2020), we rewrite the logit functions defined in (3) and (4) into a unified form

$$\begin{aligned} \textbf{C}^\mathrm{{T}}\log (\textbf{L}{\boldsymbol{\lambda }}_t)={\boldsymbol{\eta }}_t=\textbf{X}_t{\boldsymbol{\theta }}, \end{aligned}$$

where $${\boldsymbol{\eta }}_t^\mathrm{{T}}=(\eta _1(t|\textbf{x}),\ldots ,\eta _J(t|\textbf{x}),\eta _{J+1}(t|\textbf{x}))$$ and

$$\textbf{C}^\mathrm{{T}}=\left( \begin{array}{ccc} {\textbf{I}}_J& \quad -\textbf{I}_J& \quad \textbf{0}_J\\ \textbf{0}^\mathrm{{T}}_J& \quad \textbf{0}^\mathrm{{T}}_J& \quad 1 \end{array} \right) , $$

is a $(J+1)\times (2J+1)$ constant matrix, where $\textbf{I}_J$ is the identity matrix of order *J*, $\textbf{0}_J$ is a vector of *J* zeros and $\textbf{L}$ is defined as follows for the logit and cumulative model, respectively,

$$\begin{aligned} \textbf{L}_{m}=\left( \begin{array}{cccccc} 1& \quad 0& \quad 0& \quad \ldots & \quad 0& \quad 0\\ 0& \quad 1& \quad 0& \quad \ldots & \quad 0& \quad 0\\ \vdots & \quad \vdots & \quad \vdots & \quad \vdots & \quad \vdots & \quad \vdots \\ 0& \quad 0& \quad 0& \quad \ldots & \quad 1& \quad 0\\ 0& \quad 0& \quad 0& \quad \ldots & \quad 0& \quad 1\\ 0& \quad 0& \quad 0& \quad \ldots & \quad 0& \quad 1\\ \vdots & \quad \vdots & \quad \vdots & \quad \vdots & \quad \vdots & \quad \vdots \\ 0& \quad 0& \quad 0& \quad \ldots & \quad 0& \quad 1\\ 1& \quad 1& \quad 1& \quad \ldots & \quad 1& \quad 1 \end{array} \right) _{(2J+1)\times (J+1)}, \textbf{L}_c=\left( \begin{array}{cccccc} 1& \quad 0& \quad 0& \quad \ldots & \quad 0& \quad 0\\ 1& \quad 1& \quad 0& \quad \ldots & \quad 0& \quad 0\\ \vdots & \quad \vdots & \quad \vdots & \quad \vdots & \quad \vdots & \quad \vdots \\ 1& \quad 1& \quad 1& \quad \ldots & \quad 1& \quad 0\\ 0& \quad 1& \quad 1& \quad \ldots & \quad 1& \quad 1\\ 0& \quad 0& \quad 1& \quad \ldots & \quad 1& \quad 1\\ \vdots & \quad \vdots & \quad \vdots & \quad \vdots & \quad \vdots \\ 0& \quad 0& \quad 0& \quad \ldots & \quad 0& \quad 1\\ 1& \quad 1& \quad 1& \quad \ldots & \quad 1& \quad 1 \end{array} \right) _{(2J+1)\times (J+1)}. \end{aligned}$$

#### Remark 1

Estimating the parameter vector ${\boldsymbol{\theta }}$ via maximum likelihood involves optimizing over $Jq + p$ parameters, where *p* denotes the dimension of the treatment effect vector. This process can become computationally expensive as the number of inspection intervals grows. Moreover, a high-dimensional ${\boldsymbol{\theta }}$ may lead to numerical instability and compromise inferential reliability, particularly when the number of time intervals substantially exceeds the number of observed events, resulting in sparse event counts within intervals (Schmid and Berger 2021).

To mitigate these issues, several authors have proposed imposing structural constraints on the baseline coefficients $\gamma _{0tr}$. Common approaches include restricting them to follow a polynomial form (Singer and Willett 2003) or representing them via spline basis expansions (Tutz and Schmid 2016). In general, this can be expressed as:

$$ \gamma _{0tr}=\sum _{s=1}^{m_r}\gamma _{0sr}\phi _{sr}(t)={\boldsymbol{\phi }}_{r}^\mathrm{{T}}(t){\boldsymbol{\gamma }}_{0r}, r=1,2,\ldots , J. $$

where $\phi _{sr}(t)$ are pre-specified basis functions, ${\boldsymbol{\phi }}_{r}^\mathrm{{T}}(t)=(\phi _{1r}(t),\phi _{2r}(t),\ldots ,\phi _{m_rr}(t))$ is the corresponding basis vector, and ${\boldsymbol{\gamma }}_{0r}^\mathrm{{T}}=(\gamma _{01r},\gamma _{02r},\ldots ,\gamma _{0m_rr})$ is the reduced parameter vector for the baseline hazard of risk *r*. Under this representation, the design matrix for the multinomial logit model, for instance, takes the form:

$$ \textbf{X}_t=\left[ \begin{array}{ccccccccccc} {\boldsymbol{\phi }}_1^\mathrm{{T}}(t)& \quad & \quad & \quad & \quad \textbf{f}_1^\mathrm{{T}}(\textbf{x})& \quad & \quad & \quad & \quad \\ & \quad {\boldsymbol{\phi }}_2^\mathrm{{T}}(t)& \quad & \quad & \quad & \quad \textbf{f}_2^\mathrm{{T}}(\textbf{x})& \quad & \quad & \quad \\ \vdots & \quad & \quad \ddots & \quad & \quad & \quad \ddots \\ & \quad & \quad & \quad {\boldsymbol{\phi }}_J^\mathrm{{T}}(t)& \quad & \quad & \quad \textbf{f}_J^\mathrm{{T}}(\textbf{x})& \quad \\ & \quad & \quad \ldots & \quad & \quad & \quad \ldots & \quad & \quad & \quad 1 \end{array} \right] , $$

and the full parameter vector becomes ${\boldsymbol{\theta }}^\mathrm{{T}}=({\boldsymbol{\gamma }}_{01}^\mathrm{{T}},{\boldsymbol{\gamma }}_{02}^\mathrm{{T}},\ldots ,{\boldsymbol{\gamma }}_{0J}^\mathrm{{T}},{\boldsymbol{\gamma }}_{1}^\mathrm{{T}},{\boldsymbol{\gamma }}_{2}^\mathrm{{T}},\ldots ,{\boldsymbol{\gamma }}_{J}^\mathrm{{T}},0)$.

### Information matrix

Following convention, the quality of statistical inference is judged by its information matrix, which depends on the design that generates the data. Assume that we have *N* subjects in our study and each subject has *d* factors denoted by $\textbf{x}_i^{\text {T}}=(x_{i1},\cdots ,x_{id}),i=1,\ldots ,N$. At the onset, the *i*th subject is allocated to the experimental settings $\textbf{x}_i$ to collect data at the *q* time intervals $[a_{t-1},a_t), t=1,\ldots ,q$ and the response for subject *i* at the *t*th time period is a multinomial response $\textbf{Y}^{\mathrm{{T}}}_{it}=(y_{it1},\ldots ,y_{itJ},y_{it,J+1})\sim \text {M}(1;\lambda _{1}(t|\textbf{x}_i),\ldots ,\lambda _{J}(t|\textbf{x}_i),\lambda _{J+1}(t|\textbf{x}_i))$, where $\lambda _{r}(t|\textbf{x}_i)$ is the hazard rate that the response happens due to the *r*th risk under the experimental setting $\textbf{x}_i$ at the *t*th time period. We assume random censoring at the end of the interval with $t_i=\min \{T_i,C_i\}$, where $T_i$ and $C_i$ denote the event and censoring times of subject *i*. Then the log-likelihood function for the DTSM with competing risks is

7 $$\begin{aligned} \ell ({\boldsymbol{\theta }})= & \log \left( \prod _{i=1}^N\prod _{t=1}^{t_{i}}\lambda _{1}(t|\textbf{x}_i)^{y_{it1}}\cdots \lambda _{J}(t|\textbf{x}_i)^{y_{itJ}} \lambda _{J+1}(t|\textbf{x}_i)^{y_{it,J+1}}\right) \nonumber \\= & \sum _{t=1}^q\sum _{i\in R_t}\textbf{Y}_{it}^\mathrm{{T}}\log {\boldsymbol{\lambda }}_{it}, \end{aligned}$$

where $$\log ^{\mathrm{{T}}}{\boldsymbol{\lambda }}_{it}=(\log \lambda _{1}(t|\textbf{x}_i),\ldots ,\log \lambda _{J}(t|\textbf{x}_i), \log \lambda _{J+1}(t|\textbf{x}_i))$$, $$R_t=\{i|t_i\ge t\}$$ is the number of subjects at risk in the interval $$[a_{t-1},a_t)$$. Using properties of the multinomial distribution, we show in Appendix that the FIM is

8 $$\begin{aligned} \textbf{M}= & \sum _{{i}=1}^N \sum _{{t}=1}^{t_{i}}\left( \frac{\partial {\boldsymbol{\lambda }}_{it}}{\partial {\boldsymbol{\theta }}^T}\right) ^\mathrm{{T}}\left[ \operatorname {diag}({\boldsymbol{\lambda }}_{it})\right] ^{-1}\frac{\partial {\boldsymbol{\lambda }}_{it}}{\partial {\boldsymbol{\theta }}^\mathrm{{T}}}, \end{aligned}$$

where $\frac{\partial {\boldsymbol{\lambda }}_{it}}{\partial {\boldsymbol{\theta }}^\mathrm{{T}}}=\left( \textbf{C}^\mathrm{{T}}\textbf{D}_{it}^{-1}\textbf{L}\right) ^{-1}\textbf{X}_{it}$, $\textbf{D}_{it}=\operatorname {diag}(\textbf{L}{\boldsymbol{\lambda }}_{it})$ and $\textbf{X}_{it}$ is either (5) or (6).

## Optimal designs

### The design

In the previous section, we assume that there are a total of *N* subjects participating in the experiment, and they are observed over *q* time intervals $[a_0,a_1),[a_1,a_2),\ldots ,[a_{q-1},a_{q})$. The instants $\{a_0, a_1, \ldots , a_{q_{\text {max}}}\}$ constitute a fixed set of candidate inspection time points, and are pre-specified, potentially with unequal spacing, as dictated by clinical or practical considerations. Our optimal design seeks the best $q^*$ from this set. A typical example is a cohort study with follow-ups scheduled at $\{0, 1, 3, 6, 12\}$ months, reflecting periods where key clinical changes are anticipated. Our method optimizes the study duration by selecting how many of these pre-planned visits to implement. Furthermore, it is often the case that the *N* subjects are not necessarily observed under *N* different experimental conditions. Instead, we assume that there are *k* distinct experimental conditions represented by points $\textbf{x}_i$, each with a corresponding weight $\pi _i$. We denote these experimental conditions by

9 $$\begin{aligned} \xi _{\textbf{x}}=\left\{ \textbf{x}_1, \textbf{x}_2, \ldots , \textbf{x}_k; \pi _1, \pi _2,\ldots , \pi _k \right\} , \end{aligned}$$

where the support points $\textbf{x}_i$ belong to $\mathcal {X}$, the compact set of all possible values of $\textbf{x}_i$ in the problem. The weights $\pi _i$ represent the proportion of subjects with the same covariates $\textbf{x}_i$ and satisfy the constraint $\sum _{i=1}^k\pi _i=1$.

In practice, it is important to not only consider how to efficiently choose the experimental conditions $\xi _\textbf{x}$, the duration of the study, but also determine the total number of subjects. This means that we should decide the number of time periods *q* and the number of subjects *N*, taking into account the costs associated with the experiment. We use the notation $\zeta$ to represent the design, which includes all these elements:

10 $$\begin{aligned} \zeta =\left\{ \xi _\textbf{x},N, q\right\} . \end{aligned}$$

Let $n_i$ and $n_{it}, i=1,\ldots ,k, t=1,\ldots ,q$ denote, respectively, the number of subjects allocated to the *i*th experimental group, and the number of subjects entering the *t*th period of the *i*th group. Based on the design (9), the allocation of subjects can be described by $n_i = N\pi _i$. However, due to various factors, such as competition events and other factors reducing the number of test subjects, we observe that $n_{i1} \ge n_{i2} \ge \cdots \ge n_{iq}$. Moreover, at the design stage, the values of $n_{it}$ are not known and are treated as random variables due to the uncertainty associated with these factors. In what follows, we will utilize the expected number of subjects in the risk set at the beginning of the *t*th interval to replace $n_{it}$ to construct optimal designs. Specifically, we have $E\left( n_{it}\right) =n_iS(t-1|\textbf{x}_i)\prod _{s=1}^{t-1}(1-\rho _{is})$, where $S(t-1|\textbf{x}_i)$ is the survival function in $(t-1)$th time period of the *i*th group and $\rho _{is}\in [0,1]$ are attrition rates. Attrition occurs when subjects permanently leave the study for reasons unrelated to the events of interest, such as loss of follow-up or withdrawal from the study (Jóźwiak and Moerbeek 2012). For a more in-depth discussion on modeling missing data and finding optimal designs in this context, please refer to Lee et al. (2019). If the attrition rates $\rho _{is}$ at every end of the interval under different groups are equal, then we have $E(n_{it})=n_iS(t-1|\textbf{x}_i)(1-\rho )^{t-1}$. It is instructive to rewrite (8) as

11 $$\begin{aligned} \mathcal {M}(\zeta |{\boldsymbol{\theta }})= & N\sum _{{i}=1}^k \pi _i\mathcal {M}(\textbf{x}_i|{\boldsymbol{\theta }}), \end{aligned}$$

where ${\mathcal{M}}\left( {{\mathbf{x}}_{i} |{\boldsymbol{\theta}} } \right)$ $= \sum\nolimits_{{t = 1}}^{q} S \left( {t - 1|{\mathbf{x}}_{i} } \right)$ $\prod\nolimits_{{s = 1}}^{{t - 1}} {(1 - \rho _{{is}} )} {\mathbf{M}}_{{it}}$, $\textbf{M}_{it}$ $=\left( \frac{\partial {\boldsymbol{\lambda }}_{it}}{\partial {\boldsymbol{\theta }}^\mathrm{{T}}}\right) ^\mathrm{{T}}$ $\left[ \operatorname {diag}(\boldsymbol{\lambda }_{it})\right] ^{-1}\frac{\partial {\boldsymbol{\lambda }}_{it}}{\partial {\boldsymbol{\theta }}^\mathrm{{T}}}$ is the individual information matrix in the *t*th time period of the *i*th group. In what follows, we assume, for simplicity, that the attrition rates remain constant, i.e. $\rho _{is}=\rho$.

### Cost functions

It is desirable to incorporate the known costs at the design stage. We define two cost functions for different scenarios, including the case where we vary total costs across the time periods. We assume that the total budget for the study is fixed and equal to C, the setup cost is $c_0$, $c_{1i}$ is the cost of recruiting a new subject under the condition $\textbf{x}_i$ and $c_{2i}$ is the cost of obtaining a measurement per subject under condition $\textbf{x}_i$. The first cost function has the form

12 $$\begin{aligned} c_0+N\sum _{i=1}^k\pi _i\left[ c_{1i}+c_{2i}(q+1)\right] , \end{aligned}$$

where we assume that the cost of obtaining a measurement for every subject is the same under different values of $\textbf{x}$, and there is one measurement per subject in each period.

The second cost function assumes that subjects are not followed after the event occurs. This is applicable, for example, in a trial studying survival in a disease study until death. Under the design (9), the second cost function can be written as:

13 $$\begin{aligned} c_0+N\sum _{i=1}^k\pi _i\left[ c_{1i}+c_{2i}\sum _{s=0}^qS_i(a_{s})\right] , \end{aligned}$$

where $S_i(a_{s})=S(s-1|\textbf{x}_i)(1-\rho )^{s-1}$ is the survival probability of subjects under condition $\textbf{x}_i$ up to time $a_s$.

The above cost functions are illustrative and other cost structures can be used. To incorporate costs into the design optimization problem, we normalize the information matrix of a design $\zeta$ in (11) by the quantity $C-c_0$ and the standardized information matrix is

14 $$\begin{aligned} \mathcal {M}^c(\zeta |{\boldsymbol{\theta }})= \frac{\mathcal {M}(\zeta |{\boldsymbol{\theta }})}{C-c_0}=\sum _{i=1}^k \pi _i^c\frac{\mathcal {M}_i(\textbf{x}_i|{\boldsymbol{\theta }})}{c(\textbf{x}_i)}. \end{aligned}$$

Here $\pi ^c_i=\pi _ic(\textbf{x}_i)/{\sum _{i=1}^k\pi _ic(\textbf{x}_i)}$, where $c(\textbf{x}_i)=c_{1 i}+c_{2i}(q+1)$ for (12) and $c(\textbf{x}_i)=c_{1i}+c_{2i}\sum _{s=0}^qS_i(a_{s})$ for (13). Further calculation shows the relationships $\pi _i=\frac{\pi _i^c/c(\textbf{x}_i)}{\sum _{i=1}^k\pi _i^c/c(\textbf{x}_i)}$ and $N=(C-c_0)\sum _{i=1}^k\pi _i^c/c(\textbf{x}_i)$; see Fedorov and Leonov (2013) for details about the cost-based optimal design methodology.

### Design criterion

In this work, we find optimal designs for estimating all model parameters or a meaningful subset of the the model parameters. To estimate the subset, we use $D_s$-optimality as opposed to *D*-optimality for estimating all model parameters ${\boldsymbol{\theta }}$. These optimal designs minimize the volume of the confidence ellipsoids of the estimated parameters of interest, and they can be found by first selecting an appropriate matrix $\textbf{A}$ in the following design criterion before minimizing it over the set of all designs:

15 $$\begin{aligned} \Phi _{D_{\textbf{A}}}(\zeta |{\boldsymbol{\theta }})=\log |\textbf{A}^\mathrm{{T}}\left( \mathcal {M}^c(\zeta |{\boldsymbol{\theta }})\right) ^{-1}\textbf{A}|. \end{aligned}$$

For example, when the selection matrix is the identity matrix, i.e., $\textbf{A}=\textbf{I}_{qJ+p+1}$, the resulting design $\zeta ^*$ is a *D*-optimal design for estimating the entire parameter vector. Here, *p* represents the total dimension of the treatment effects, defined as $p=\dim ({\boldsymbol{\gamma }}_1)+\ldots +\dim ({\boldsymbol{\gamma }}_J)$ or simply $\dim ({\boldsymbol{\gamma }})$, depending on whether a multinomial logit model or cumulative logit model is considered. Conversely, if $\textbf{A}$ is the $(qJ+p+1)\times p$ matrix formed by stacking the matrices $\textbf{0}_{qJ\times p }$, $\textbf{I}_{p\times p}$ and $\textbf{0}_{1\times p}$, then the design $\zeta ^*$ becomes $D_s$-optimal. This $D_s$-criterion minimizes the generalized variance of the treatment effect $(\boldsymbol{\gamma }_{1}^\mathrm{{T}}, \ldots , \boldsymbol{\gamma }_{J}^\mathrm{{T}})^T$ or $\boldsymbol{\gamma }$, which is a sub-vector of model parameters ${\boldsymbol{\theta }}$.

#### Remark 2

While this paper primarily focuses on the *D*- and $D_s$-optimal design criteria for methodological simplicity, we recognize that in survival analysis with competing risks, researchers often target the CIF for the *r*th event type as the primary inferential quantity (Schmid and Berger 2021). It is important to note that the CIF (defined in Eq. (2)) is a function of the parameter vector ${\boldsymbol{\theta }}$. Therefore, after obtaining a maximum likelihood estimate of ${\boldsymbol{\theta }}$, we can substitute it into the CIF to obtain $\hat{F}_{r}(t | \textbf{x})$, which represents the estimated CIF. The asymptotic covariance matrix of this estimator can be derived via the Delta method as:

$$ \textrm{Cov}\left( \hat{F}_{r}(t | \textbf{x})\right) \approx \left( \frac{\partial F_{r}(t | \textbf{x})}{\partial \boldsymbol{{\boldsymbol{\theta }}}}\right) ^\textrm{T} \mathcal {M}(\zeta |{\boldsymbol{\theta }})^{-1} \frac{\partial F_{r}(t | \textbf{x})}{\partial \boldsymbol{{\boldsymbol{\theta }}}}. $$

By substituting $\mathcal {M}(\zeta |\boldsymbol{\theta })^{-1}$ with $\mathcal {M}^c(\zeta |\boldsymbol{\theta })^{-1}$ and then minimizing the asymptotic variance with respect to the design $\zeta$, we can obtain the *c*-optimal design under a cost constraint (Fedorov and Leonov 2013).

To fairly compare the optimal design $\zeta ^*$ with another design $\zeta$ under the same setup, we use its relative efficiency (RE) given by

16 $$\begin{aligned} \textrm{RE}_{D_A}(\zeta ^*|\zeta )=\frac{\left| \textbf{A}^\mathrm{{T}}\left( \mathcal {M}^c(\zeta ^*|{\boldsymbol{\theta }})\right) ^{-1}\textbf{A}\right| ^{\frac{1}{v}}}{\left| \textbf{A}^\mathrm{{T}}\left( \mathcal {M}^c(\zeta |{\boldsymbol{\theta }})\right) ^{-1}\textbf{A}\right| ^{\frac{1}{v}}}, \end{aligned}$$

where *v* is the rank of $\textbf{A}$. If the ratio is equal to 0.5, this means that the design $\zeta$ needs to replicated twice to perform as well as the optimal design $\zeta ^*$.

## Sensitivity analysis

The design criterion in (15) contains many parameters, and their relative influence on the optimal design is unknown, potentially varying with the statistical model, the optimality criterion, and the design space. To make this problem tractable, we concentrate on a two-arm randomized trial and consider both the presence and absence of time-varying treatment effects. We derive locally optimal designs Fedorov and Leonov (2013), which requires pre-specifying best-guess values for the model parameters ${\boldsymbol{\theta }}$. This focused approach allows us to obtain concrete results and insights into the design’s behavior under these specified conditions. However, as discussed in Remark 1, the dimension of the baseline coefficients $\gamma _{0tr}$ becomes large with increasing inspection intervals, posing a significant challenge for parameter specification in sensitivity analysis. Consequently, to alleviate this difficulty, we adopt the approach advocated by Moerbeek and her collaborators (Jóźwiak and Moerbeek 2012, 2013; Moerbeek and Wong 2015; Safarkhani and Moerbeek 2015). This method posits that the underlying survival times are continuous and can be fitted using a parametric continuous-time survival model. By equating the hazard rates from this continuous-time model to the corresponding discrete-time baseline hazards (where no covariate effects are considered), we transform the challenging task of directly setting the baseline parameters in the original discrete-time model into the more straightforward problem of selecting parameters for the continuous-time model. We extend their method to the competing risks setting, with a detailed description provided in the following subsection.

### Parametric competing risks models

Over the past few decades, the methodological toolkit for continuous-time competing risks analysis has expanded significantly. Key advancements include non- and semi-parametric models such as the cause-specific hazards model and the Fine–Gray model (Fine and Gray 1999), as well as direct models for absolute risk (Gerds et al. 2012). In addition to these non-parametric and semi-parametric approaches, several parametric formulations have been developed, including mixture models (Lau et al. 2008) and restricted parametric models (Shi et al. 2013). Concurrently, machine learning techniques are increasingly being explored as alternative methodologies. For a comprehensive review and comparison of these diverse approaches, refer to Monterrubio-Gómez et al. (2024). To simplify the process of setting the nominal values for the baseline coefficients in our design criterion, we restrict our focus to parametric models with competing risks. We assume that the CIF $F_r(t | \textbf{x})$ for *r*th risk can be expressed using the transformation model: $g_r(F_r(t | \textbf{x})) = h_{r0}(t) + \textbf{f}_r^\textrm{T}(\textbf{x}) \boldsymbol{\gamma }_r, \quad r = 1, \ldots , J,$ with the constraint that $\sum _{r=1}^J g_r(F_r(+\infty | \textbf{x})) = 1$ (Shi et al. 2013). Here, $g_r$ represents known non-decreasing functions that may take on different forms, and $h_{r0}(t)$ is monotone increasing function (Fine and Gray 1999; Shi et al. 2013; Lee 2022). Without loss of generality, we assume that there are two competing risks in the trial, i.e., $J = 2$, in the remainder of this paper. Additionally, we postulate that $h_{r0}(t)$ depends on a conditional Weibull distribution, which enables flexible modeling of a hazard function that can be decreasing, increasing, or remain constant over time (Zhou et al. 2021).

Recall that the Weibull survival function is defined by $S(t)=e^{-\lambda t^{\tau }}$, where $\lambda$ and $\tau$ are the scale and shape parameters, respectively, and its hazard function is $\lambda (t)=\lambda \tau t^{\tau -1}$. Let $g_1(v)=\log (-\log (1-v))$, which represents the complementary log-log link function. We define $h_{01}(t)=g_1(\kappa (1-\exp (-\lambda _1 t^{\tau _1})))$, where the parameter $\kappa$ controls the upper asymptote of the CIF for risk 1, and corresponds to the long-term probability of the risk 1 event, as $F_1(t|\textbf{0})\rightarrow \kappa$ when $t\rightarrow \infty$. Consequently, the CIF for the first risk is given by $F_1(t|\textbf{x})$ $1-\left[ 1-\kappa \left( 1-\exp (-\lambda _1 t^{\tau _1}) \right) \right] ^{\exp ({\textbf{f}_1^\textrm{T}(\textbf{x}){\boldsymbol{\gamma }}_1})}$. Let $g_2(v)$ $= \log \left[ -\log \left( 1 - \frac{v}{1 - F_1(\infty | \textbf{x})}\right) \right]$ and $h_{02}(t) = g_1(1 - \exp (-\lambda _2 t^{\tau _2}))$. Then, the CIF for the second risk is given by: $F_2(t | \textbf{x})$ $= \left\{ 1 - \exp \left[ -\lambda _2 t^{\tau _2} \exp (\textbf{f}_2^\textrm{T}(\textbf{x}) \boldsymbol{\gamma }_2)\right] \right\}$ $(1 - \kappa )^{\exp (\textbf{f}_1^\textrm{T}(\textbf{x}) \boldsymbol{\gamma }_1)}$. Special cases for $\lambda _r = 1$ and $\tau _r = 1$ can be found in Fine and Gray (1999) and Lee (2022). When $\textbf{x} = \textbf{0}$, we have $F_1(t | \textbf{0}) = \kappa \left( 1 - \exp (-\lambda _1 t^{\tau _1})\right)$ and $F_2(t | \textbf{0}) = (1 - \kappa ) \left( 1 - \exp (-\lambda _2 t^{\tau _2})\right) .$ This mixture distribution framework for characterizing the CIFs was previously established by Cheng (2009). Note that replacing the scale parameter $\lambda _r$ by $-\log (1-w_r)$ with $w_r\in (0,1)$, we can re-parameterize the CIFs $F_r(t|\textbf{x}),r=1,2$ defined above. Here, the parameter $w_r$ can represent the proportion of subjects that have experienced the events due to risk *r* at $t=1$ (Moerbeek and Wong 2015; Zhou et al. 2021).

Let $\lambda _r(t \mid \textbf{0})$ denote the baseline discrete-time hazard probability for the *r*th risk in time period *t*, defined as: $\lambda _r(t \mid \textbf{0})$ $= P(T = t, R = r \mid T \ge t, \textbf{x} = \textbf{0})$ $= {(F_r(a_t \mid \textbf{0}) - F_r(a_{t-1} \mid \textbf{0}))}/{(1 - F_r(a_{t-1} \mid \textbf{0}))}$. This definition implies that the values of the baseline coefficients $\gamma _{0tr}$ are determined by the discrete-time hazard probabilities derived from the cumulative incidence function (CIF) for the *r*th event when $\textbf{x} = \textbf{0}$. Consequently, the design criterion $\Phi _{D_{\textbf{A}}}(\zeta \mid \boldsymbol{\theta })$ ultimately depends only on the Weibull distribution parameters $\tau _r$ (shape) and $\lambda _r$ (scale) for the *r*th event, along with the proportion parameter $\kappa$. In the subsequent analysis, we use the multinomial logit model to illustrate our design methodology for a two arm trial with or without time-varying treatment effects.

### Optimal designs for placebo-treatment comparison with time-independent treatment effects

We investigate a two-arm longitudinal trial design that compares an experimental treatment ($x=1$) to a control ($x=0$), where subjects may experience one of two competing events. The cause-specific hazards are modeled via:

17 $$\begin{aligned} \eta _r(t|x)=\gamma _{0tr}+\gamma _{r1}x, r=1,2, \quad t=1,\ldots ,q, \end{aligned}$$

with the design space restricted to $\mathcal {X}=\{0,1\}$. Optimal designs for the model in Eq. (17) with a single target event have been thoroughly addressed by Jóźwiak and Moerbeek (2012, 2013). In this context, our interest lies in finding a design that best estimates the treatment effects $\gamma _{11}$ and $\gamma _{21}$, with $D_s$-optimality serving as the design criterion. To overcome the dependence of the design criterion on the parameters in model (17), we first calculate the hazard probabilities in the control group via an underlying continuous time Weibull function, before obtaining the values for the parameters $\gamma _{0t1}, \gamma _{0t2}, t=1,2,\ldots , q$. The model parameters are set as follows: $w_1, w_2\in \{0.3,0.5\}$, $\tau _1,\tau _2\in \{1/3,3\}$, $\kappa \in \{0.3,0.5,0.7\}$ and $\gamma _{11},\gamma _{21}\in \{-2.5,2.5\}$. The cost ratio, defined as $f_i = c_{1i}/c_{2i}$, takes values in $\{1, 50, 100\}$.

The goal is to construct an optimal design for a 12-month trial for estimating the two treatment parameters. A design question is the choice of the optimal number of time periods to observe the outcomes. For example, would a trial with 6 or 12 time periods be more efficient? In our work, we investigate the impact of having 6 or 12 time periods on the estimates of the survival and hazard probabilities in the study.

**Fig. 1:**
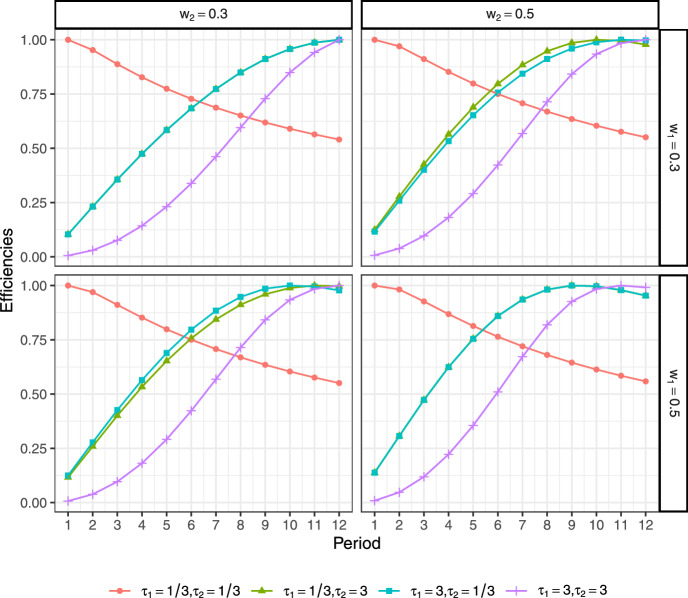
Efficiency lines for different $(w_r,\tau _r), r=1,2$ with cost ratio $f=1$, treatment effects $\gamma _{11}=2.5,\gamma _{21}=2.5$, proportion parameter $\kappa =0.5$, and attrition rate $\rho =0$

We first consider trials with two equally sized groups (Jóźwiak and Moerbeek 2012) and assume that the second cost function (13) is appropriate. From the above statements, the design in Eq. (10) simplifies to $\zeta =\{q\}$ and there is only one variable in the design to be optimized. Accordingly, we calculate optimal time designs (periods) under different parameter settings and the corresponding efficiencies of the optimal designs. Table 1 presents optimal time periods for different parameter settings. For illustration, Fig. 1 plots the efficiency lines with different $w_r$ and $\tau _r$, $r=1,2$ when the cost ratio $f=f_1=f_2=1$, the proportion parameter $\kappa =0.5$ and treatment effects $\gamma _{11}=2.5,\gamma _{21}=2.5$. This figure indicates that the shape parameter $\tau$ in the Weibull distribution has a greater effect on the optimal design than the scale parameter *w*, primarily because it dictates the temporal pattern of the hazard. Specifically, for small $\tau _r$ ($r=1,2$,e.g., $<1$), the decreasing hazard function leads to an optimal design $q^*$ focused on the beginning of the trial (i.e., optimal inspection times near 1), where events are most prevalent. Conversely, for large $\tau _r$ ($r=1,2$, e.g., $>1$), the increasing hazard function necessitates a design where $q^*$ is concentrated toward the end of the study period (i.e., optimal inspection times near 12), where the risk is highest. Moreover, the nominal values of the treatment effects $\gamma _{r1}, r=1,2$ will also influence the optimal designs (see Table 1). In general, as the values of $\gamma _{r1}, r=1,2$ increase, the optimal number of time periods $q^*$ will either remain constant or decrease. Except for the values of the parameters $\tau ,w$ and $\gamma _{r1}$, the cost ratio *f* and the attrition rate $\rho$ will also affect the optimal time periods. When the cost ratio *f* or $\rho$ increases, the optimal $q^*$ tends to increase (see Tables S1 and S2 in Web Appendix A). This implies that as the cost ratio of recruiting an individual to obtaining a measurement increases, or as the attrition rate at each time period increases, the duration of the trial tends to become longer. Furthermore, we examine the impact of the proportion parameter $\kappa$ on the optimal time periods (refer to Tables S3 and S4 in Web Appendix A), which shows that the design is robust to this parameter, with minimal impact observed.

**Table 1:** D_s-optimal designs $\zeta ^*=\{ q^*\}$ for model (17) under varying parameters $w_r,\tau _r, \gamma _{r1}, r=1,2$ with $\kappa =0.5$, $f=1$, $\rho =0$, and equal weights allocated to the two groups

$\tau _1$	$\tau _2$	$w_1$	$w_2$	$(\gamma _{11},\gamma _{21})$			
				(−2.5,−2.5)	(−2.5,2.5)	(2.5,−2.5)	(2.5,2.5)
1/3	1/3	0.3	0.3	1	1	1	1
1/3	1/3	0.3	0.5	1	1	1	1
1/3	1/3	0.5	0.3	1	1	1	1
1/3	1/3	0.5	0.5	1	1	1	1
1/3	3	0.3	0.3	12	12	12	12
1/3	3	0.3	0.5	12	12	12	10
1/3	3	0.5	0.3	12	12	12	11
1/3	3	0.5	0.5	12	12	12	9
3	3	0.3	0.3	12	12	12	12
3	3	0.3	0.5	12	12	12	12
3	3	0.5	0.3	12	12	12	12
3	3	0.5	0.5	12	12	12	11

So far, we have studied optimal designs for trials with two treatment groups that are of equal size at the beginning of the trials. However, this may not necessarily be the optimal choice (Jóźwiak and Moerbeek 2013). In the subsequent part of this subsection, we relax the restriction of equal group sizes and explore optimal designs with possible unequal group sizes. In this case, the design defined in (10) simplifies to $$\zeta =\{\pi ,q\}$$, where $$\pi $$ is the proportion of subjects allocated to the experimental group. To find the optimal design $$\zeta ^*$$, we discretize the weight space [0, 1] into 100 equal intervals, resulting in a total of 1188 possible designs. The optimal design $$\zeta ^*$$ is the one that minimizes $$\Phi _{D_A}(\zeta |{\boldsymbol{\theta }})$$ among these possible designs. Table 2 presents optimal designs $$\zeta ^*$$ for different parameter settings. Similar conclusions regarding the optimal number of time periods can be derived from the optimal designs with unequal group sizes, as those obtained from the optimal designs with equal sizes. The optimal weights $$\pi ^*$$ are primarily influenced by the parameters $$\gamma _{r1}, r=1,2$$, in comparison to other parameters. Specifically, when the values of $$\gamma _{r1}, r=1,2$$ are small, the corresponding optimal weights $$\pi ^*$$ tend to be large. In addition, we also calculate the efficiencies of the optimal design with equal group sizes, and present them in the RE columns. Again, the parameters $$\gamma _{r1}, r=1,2$$ play an important role. As the values of $$\gamma _{r1}, r=1,2$$ decrease, the efficiency of the optimal designs with equal group sizes also decreases. Similar results can be found for optimal designs when the parameter *f* or $$\rho $$ become large (see Tables S5 and S6 in Web Appendix A). We observe that, in general, optimal designs with equal group sizes tend to perform well in most situations compared to optimal designs that do not have size restrictions. However, it is important to note that there are certain situations where allocating subjects equally to different groups may not be considered favorable. This is particularly true when the values of the parameters $$\gamma _{r1}$$, $$ r=1$$ or 2, tend to be small, indicating lower hazard rates. The practical implication is that when the hazard rates in all groups are low, the equal allocation strategy may exhibit poor performance. Figures S1–S3 presented in Web Appendix A provide additional evidence. Furthermore, consistent with the findings from designs with equal group sizes, the parameter $$\kappa $$ is also observed to have a negligible influence on the optimal designs even when group size constraints are relaxed (see Tables S7 and S8 in Web Appendix A). This indicates that the optimal design is robust to variations in $$\kappa $$.

**Table 2:** $D_s$-optimal designs $\zeta ^*=\{\pi ^*, q^*\}$ for model (17) under varying parameters $w_r, \tau _r, \gamma _{r1}, \, r=1,2$ with a cost ratio of $f=1$, a proportion parameter of $\kappa =0.5$, and an attrition rate of $\rho =0$

$\tau _1$	$\tau _2$	$w_1$	$w_2$	$(\gamma _{11},\gamma _{21})$											
				(−2.5,−2.5)			(−2.5,2.5)			(2.5,−2.5)			(2.5,2.5)		
				$\pi ^*$	$q^*$	RE	$\pi ^*$	$q^*$	RE	$\pi ^*$	$q^*$	RE	$\pi ^*$	$q^*$	RE
1/3	1/3	0.3	0.3	0.76	1	0.79	0.56	1	0.99	0.56	1	0.99	0.40	1	0.94
1/3	1/3	0.3	0.5	0.75	1	0.80	0.60	1	0.97	0.56	1	0.99	0.44	1	0.97
1/3	1/3	0.5	0.3	0.75	1	0.80	0.56	1	0.99	0.60	1	0.97	0.44	1	0.97
1/3	1/3	0.5	0.5	0.74	1	0.81	0.60	1	0.97	0.60	1	0.97	0.47	1	0.99
3	1/3	0.3	0.3	0.76	12	0.79	0.54	12	1.00	0.57	12	0.99	0.41	12	0.97
3	1/3	0.3	0.5	0.76	12	0.79	0.57	12	0.99	0.57	12	0.99	0.42	10	0.97
3	1/3	0.5	0.3	0.75	12	0.80	0.53	12	1.00	0.61	12	0.96	0.48	11	1.00
3	1/3	0.5	0.5	0.75	12	0.81	0.56	12	0.99	0.61	12	0.96	0.48	9	1.00
3	3	0.3	0.3	0.76	12	0.79	0.55	12	0.99	0.55	12	0.99	0.38	12	0.95
3	3	0.3	0.5	0.75	12	0.80	0.59	12	0.98	0.55	12	0.99	0.40	12	0.97
3	3	0.5	0.3	0.75	12	0.80	0.55	12	0.99	0.59	12	0.98	0.40	12	0.97
3	3	0.5	0.5	0.75	12	0.81	0.59	12	0.98	0.59	12	0.98	0.41	11	0.97

Here $\pi ^*$ denotes the optimal proportion allocated to the experimental group

### Optimal designs for placebo-treatment comparison with time-varying treatment effects

In Sect. 4.2, we make the assumption that the treatment effects remain constant across all time periods of the study. However, in practice, it is possible for the differences between treatment groups to either increase or decrease over time. Safarkhani and Moerbeek (2015) have provided examples illustrating this phenomenon. In the following of this subsection, we address the design issue for the DTSM with two competing risks in the presence of linearly divergent treatment effects. The model is described as follows:

18 $$\begin{aligned} \eta _r(t|x)=\gamma _{0tr}+\gamma _{r1}x+\gamma _{r2}x(t-1), r=1,2,\quad t=1,\ldots ,q. \end{aligned}$$

Here, $\gamma _{11}$ and $\gamma _{21}$ represent the effects of the treatment on the interested events 1 and 2, respectively. The parameters $\gamma _{12}$ and $\gamma _{22}$ capture the linear changes in treatment effects across consecutive time periods. The values of the parameters are determined as in Sect. 4.2. In addition, the parameters $\gamma _{11}, \gamma _{21}$ are assumed to be 0.3, and $\gamma _{12}$ and $\gamma _{22}$ are chosen from the set $\{-0.5,0, 0.5\}$. It is worth noting that when either $\gamma _{12}$ or $\gamma _{22}$ is set to 0, it implies that the effect of the treatment for one of the interested events is independent of the observed time periods.

We consider various design criteria for different design aims: *D*-optimality for estimating all model parameters ${\boldsymbol{\theta }}$, and two types of $D_s$-optimality for estimating two subsets of ${\boldsymbol{\theta }}$; $D_{s_1}$-optimality for estimating the treatments effects $\gamma _{rj}, r=1,2, j=1,2$ and $D_{s_2}$-optimality for estimate the treatment effects $\gamma _{1j}, j=1,2$. These optimal designs can be identified by first selecting an appropriate matrix $\textbf{A}$ in Eq. (15) to be minimized. Table 3 presents *D*-optimal designs $\zeta ^*=\{\pi ^*, q^*\}$ and the efficiencies of the optimal design with equal group sizes for different parameter settings, assuming $w_r=0.3$ and $\rho =0$. The results suggest that the parameters $\tau _r$ have a significant impact on the optimal time periods $q^*$. As $\tau _r, r=1,2$ increase, the optimal values of $q^*$ tend to increase. Similarly, as the cost ratios $f_i, i=1,2$ increase, the optimal values of $q^*$ tend to increase. The parameters $\gamma _{r2}, r=1,2$ also affect the optimal design $\zeta ^*$. In most cases, increasing $\gamma _{r2}, r=1,2$ from − 0.5 to 0.5 results in an increase in $\pi ^*$, while $q^*$ either decreases or remains constant. Table 3 also implies that the optimal designs with equal group sizes perform very well in all cases. Similar results can be obtained for the case $w_r=0.5$. For subset estimation, Tables S9 and S10 presented in Web Appendix A display the optimal designs $\zeta ^*=\{\pi ^*, q^*\}$ and the efficiencies of the optimal designs with equal group sizes for the $D_{s_1}$-optimality and $D_{s_2}$-optimality, respectively. We observe that most conclusions derived from *D*-optimality also hold true for $D_{s_1}$-optimality and $D_{s_2}$-optimality. Additionally, the optimal trial duration under $D_{s_1}$-optimality or $D_{s_2}$-optimality tend to be longer compared to their corresponding optimal designs under *D*-optimality.

**Table 3:** D-optimal designs $$\zeta ^*=\{\pi ^*, q^*\}$$ and the relative efficiencies of the optimal designs with equal group sizes for various $$\tau _r$$, $$\gamma _{r2}, r=1,2$$ and $$f_i, i=1,2$$ when $$\gamma _{11}=\gamma _{21}=0.3$$, $$w_r=0.3$$, $$ r=1,2$$, $$\kappa =0.5$$ and $$\rho =0$$

$f_1$	$f_2$	$\tau _1$	$\tau _2$	$\gamma _{12}$	$\gamma _{22}=-0.5$			$\gamma _{22}=0$			$\gamma _{22}=0.5$		
					$\pi ^*$	$q^*$	RE	$\pi ^*$	$q^*$	RE	$\pi ^*$	$q^*$	RE
1	1	1/3	1/3	−0.5	0.50	2	1.00	0.50	2	1.00	0.50	2	1.00
				0.5	0.50	2	1.00	0.50	2	1.00	0.50	2	1.00
		1/3	3	−0.5	0.49	3	1.00	0.48	4	1.00	0.51	5	1.00
				0.5	0.54	5	1.00	0.69	9	0.96	0.75	8	0.91
		3	3	−0.5	0.18	12	0.85	0.32	12	0.97	0.48	10	1.00
				0.5	0.48	10	1.00	0.67	9	0.97	0.76	9	0.91
1	100	1/3	1/3	−0.5	0.50	2	1.00	0.49	3	1.00	0.51	3	1.00
				0.5	0.51	3	1.00	0.53	3	1.00	0.73	7	0.95
		1/3	3	−0.5	0.21	10	0.95	0.33	10	0.97	0.49	8	1.00
				0.5	0.51	11	1.00	0.69	11	0.96	0.77	9	0.90
		3	3	−0.5	0.18	12	0.85	0.32	12	0.97	0.39	12	0.98
				0.5	0.39	12	0.98	0.64	10	0.99	0.76	9	0.92
100	100	1/3	1/3	−0.5	0.48	3	1.00	0.49	3	1.00	0.51	3	1.00
				0.5	0.51	3	1.00	0.56	4	1.00	0.73	7	0.94
		1/3	3	−0.5	0.17	12	0.92	0.29	12	0.96	0.48	9	1.00
				0.5	0.51	11	1.00	0.69	11	0.96	0.77	9	0.90
		3	3	−0.5	0.18	12	0.85	0.32	12	0.97	0.39	12	0.98
				0.5	0.39	12	0.98	0.64	10	0.99	0.76	9	0.92

Here, $\pi ^*$ denotes the optimal proportion allocated to the experimental group

**Fig. 2:**
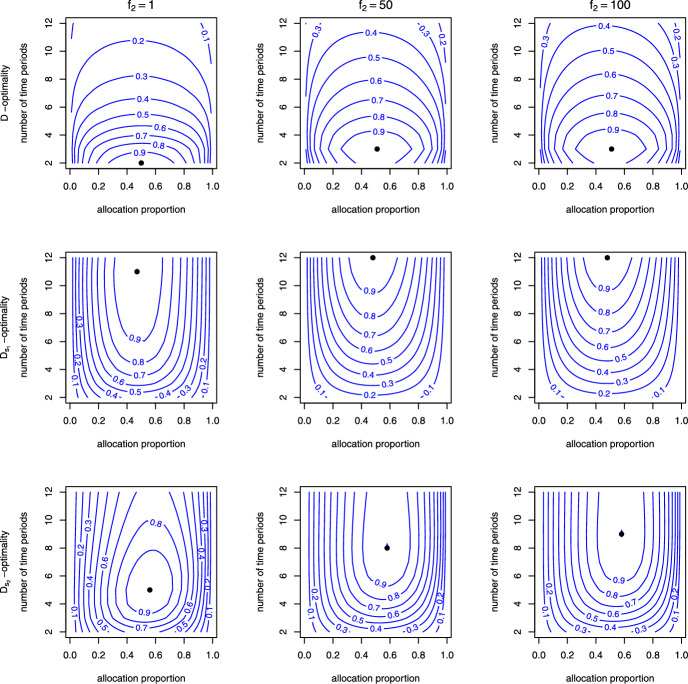
Relative efficiencies as functions of *q*, the number of time periods, and $\pi$, the allocation proportion, with different cost ratios and design criteria, for the case $\gamma _{11}=0.3,\gamma _{12}=-0.5, \gamma _{21}=0.3,\gamma _{22}=0.5$, $(w_1,\tau _1)=(0.3,1/3), (w_2,\tau _2)=(0.5,1/3)$, $\rho =0$ and $f=1$

Figure  2 plots the contour lines for the efficiency functions of the number of time periods *q* and the allocation proportion $\pi$ to the experimental group with different cost ratios $f_2$ (column in matrix of plots) and design criteria (row in matrix of plots) when $\gamma _{11}=0.3,\gamma _{12}=-0.5, \gamma _{21}=0.3,\gamma _{22}=0.5, (w_1,\tau _1)=(0.3,1/3), (w_2,\tau _2)=(0.5,1/3), \rho =0$ and $f_1=1$. In each plot, the contour lines represent a set of alternative designs $\zeta =\{\pi ,q\}$ that have the same relative efficiency (RE) values of $0.1, 0.2,\ldots ,$ or 0.9. The optimal designs are indicated by dots on the plot. In each graph, the region bounded by the contour line with RE = 0.8 encompasses all the suboptimal designs with a RE equal to 0.8 or higher. Figure 2 illustrates that the optimal proportions $\pi$ in the optimal designs tend to be close to 0.5, while the optimal number of time periods depends on the design criteria and the cost ratio $f_2$. When considering *D*-optimality, the optimal $q^*$ values tend to be concentrated at the beginning of the trial, resulting in lower efficiencies when compared to the other two design criteria. Similarly, optimal designs derived from $D_{s_1}$-optimality and $D_{s_2}$-optimality exhibit poorer performance in terms of *D*-optimality. Moreover, the contour lines for *D*-optimality have a different shape compared to the other two design criteria. The *D*-optimal design criterion is more sensitive to the choice of *q* compared to the other two design criteria. Additionally, the area enclosed by RE=0.8 or RE=0.9 is smaller for *D*-optimality compared to the other two design criteria. If the baseline hazard probability functions increase over time ($\tau _r>1$), it indicates that the target events are more likely to occur towards the end of the study. In such cases, conducting more measurements leads to higher efficiency (see Fig. S4 in Web Appendix A).

## Application

The SANAD (Standard and New Antiepileptic Drugs) study, conducted by Marson et al. (2007), is a pivotal randomized controlled trial designed to compare the long-term clinical outcomes of standard and new antiepileptic drugs (AEDs). This study specifically evaluated the efficacy of carbamazepine (CBZ), a longstanding standard treatment, against the newer drug lamotrigine (LTG). The dataset *epileptic* in R package joineR comprises longitudinal measurements of calibrated doses for participants randomized to either CBZ or LTG, along with baseline covariates and information on the time to withdrawal from the assigned treatment due to treatment failure. The primary analysis, which focused on patients with partial epilepsy, found that LTG had a significantly lower treatment failure rate compared to CBZ. Notably, LTG demonstrated favorable outcomes regarding withdrawals due to inadequate seizure control (ISC), while not being significantly worse for patients who discontinued due to unacceptable adverse effects (UAE). To investigate the impact of drug titration on the relative effects of LTG and CBZ on treatment failure, Williamson et al. (2008) employed a joint modeling approach to reanalyze the SANAD trial data, treating ISC and UAE as two competing risks. Their analysis indicated that LTG remained the preferred AED even after accounting for the effects of drug titration.

In the dataset *epileptic*, a total of 605 patients were recorded, with 292 allocated to CBZ and 313 allocated to LTG. Throughout the trial, 94 patients withdrew from the randomized drugs due to unacceptable adverse effects (UAE), while 120 withdrew due to inadequate seizure control (ISC). The times of drug withdrawal for patients were measured on a discrete scale (in days) with a range of 13 to 2400 days. To apply the discrete-time survival model with competing risks discussed in this paper, we convert the withdrawal times from days into months, using $t = 1, 2, \ldots , 80$. For the reanalysis of the rescaled data, we focus on ISC as the primary event and treat UAE as the competing risk. Given the numerous time periods involved in this application, we specify the baseline coefficients as a second-order polynomial function, as recommended by Singer and Willett (2003), using the AIC and BIC criteria for model selection. This leads to the formulation: $\gamma _{0tr} = \gamma _{00r} + \gamma _{01r} t + \gamma _{02r} t^2, r = 1, 2.$ See Remark 1 for further discussion. Under this formulation, the linear predictor can be expressed as:

19 $$\begin{aligned} \eta _r(t|x) = \gamma _{00r} + \gamma _{01r} t + \gamma _{02r} t^2 + \gamma _{r1} x, \quad r = 1, 2. \end{aligned}$$

**Fig. 3:**
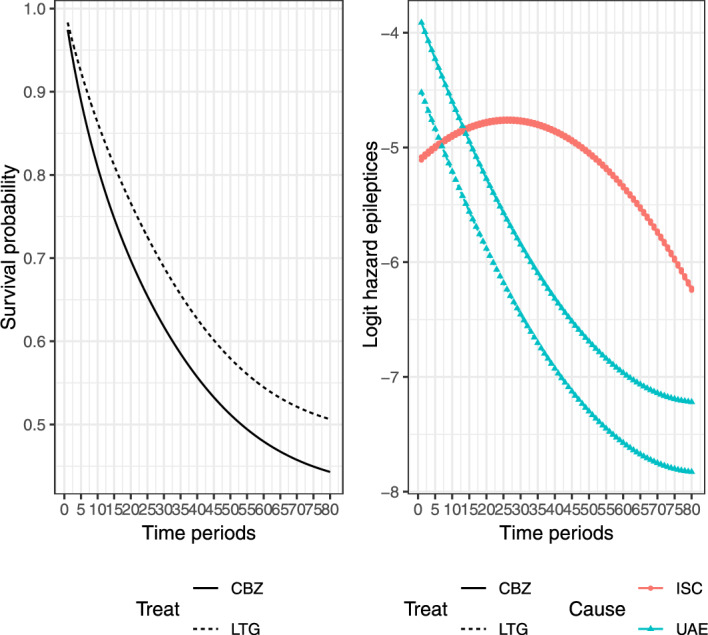
Fitted survival and logit hazard functions for different treatments using model (19) in the SANAD study

To keep with the scenario we consider in this paper, a multinomial logit model with only the covariate ‘Treat’ is fitted. The covariate ‘Treat’ takes a value of 1 if the patient is assigned to LTG and 0 otherwise. The fitted procedure is implemented by the R package VGAM. The estimated parameters are $\hat{\gamma }_{001} = -5.116$, $\hat{\gamma }_{011} = 2.128$, $\hat{\gamma }_{021} = -3.225$, $\hat{\gamma }_{11} = 0.01854$, $\hat{\gamma }_{002} = -3.825$, $\hat{\gamma }_{012} = -6.550$, $\hat{\gamma }_{022} = 3.158$, and $\hat{\gamma }_{21} = -0.60927$. The survival and logit hazard functions are plotted in Fig. 3. Obviously, this indicates that the risk function is inconsistent with the corresponding risk function of the Weibull distribution.

In the sequel of this section, we redesign the longitudinal study to investigate the relative effects of LTG and CBZ on treatment failure when it is inappropriate to describe the competing risks using the Weibull distributions. Note that for model (19), the matrix $\textbf{X}_t$ and the parameter vector ${\boldsymbol{\theta }}$, as discussed in Sect. 2.3 can be specified as

$$ \textbf{X}_t= \begin{bmatrix} 1& \quad t& \quad t^2& \quad 0& \quad 0& \quad 0& \quad 1& \quad 0& \quad 0\\ 0& \quad 0& \quad 0& \quad 1& \quad t& \quad t^2& \quad 0& \quad 1& \quad 0\\ 0& \quad 0& \quad 0& \quad 0& \quad 0& \quad 0& \quad 0& \quad 0& \quad 1 \end{bmatrix}, $$

and ${\boldsymbol{\theta }}^\mathrm{{T}}=(\gamma _{001},\gamma _{011},\gamma _{021},\gamma _{002},\gamma _{012},\gamma _{022},\gamma _{11},\gamma _{21},0)$. We assume that the maximum number of intervals is 80. Since the original study did not report the costs of collecting the dataset, we consider different cost ratios $f=f_i=1,100, i=1,2$. Furthermore, we consider the impact of different costs functions, design criteria (*D*-optimality and $D_s$-optimality), attrition rate ( $\rho =0$ and $\rho =0.2$) on the optimal designs. In addition, we also calculate efficiencies of the optimal designs $\zeta _{eq}$ with equal group sizes and the original design $\zeta _{ori}= \{\xi _x,605,80\}$ with $\xi _x=\{0,1;292/605, 313/605\}$, where 0 refers to the CBZ treatment and 1 refers to the LTG treatment. The R code for implementing the optimal design calculations is provided in the Supplementary Material. Table 4 shows the optimal weights and inspection time periods and their criterion values. Meanwhile, we also give the efficiencies of the optimal designs with equal group sizes in the 8th column and the efficiencies of the original design in the 9th column in Table 4. The results indicate that *D*-optimal designs favor running the trial for the longest duration possible when the attrition rate is zero, as more observations provide additional information for efficiently estimating all parameters in Model (19). However, when treatment effects are of interest, the duration of the $D_s$-optimal designs is shorter than or equal to that of their corresponding *D*-optimal designs. Furthermore, the optimal weights of the $D_s$ designs tend to allocate more weight to the LTG group. In addition, Table 4 shows that the optimal designs with equal group sizes perform consistently well across all scenarios, whereas the effectiveness of the original designs varies depending on the design criteria, the cost function, and other factors.

**Table 4:** Optimal designs $\zeta ^*=\{\pi ^*,q^*\}$ for the model (19) and the efficiencies of the optimal design with equal group sizes, $\zeta _{eq}$ and the original design $\zeta _{ori}$ for the SANAD study

Criterion	Cost function	*f*	$\rho$	$\pi ^*$	$q^*$	$\Psi _{D_A}$	$RE_{D_A}(\zeta _{eq}|\zeta ^*)$	$RE_{D_A}(\zeta _{ori}|\zeta ^*)$
*D*	1	1	0.0	0.46	80	8.125	0.999	0.997
	2	1	0.0	0.46	80	7.671	0.999	0.997
	1	100	0.0	0.46	80	8.917	0.999	0.997
	2	100	0.0	0.46	80	8.736	0.999	0.997
	1	1	0.2	0.45	16	10.767	0.997	0.338
	2	1	0.2	0.45	80	9.174	0.998	0.996
	1	100	0.2	0.45	32	12.385	0.998	0.771
	2	100	0.2	0.45	80	12.092	0.998	0.996
$D_{s}$	1	1	0.0	0.53	9	7.143	0.996	0.467
	2	1	0.0	0.53	9	6.980	0.996	0.624
	1	100	0.0	0.53	54	8.642	0.997	0.946
	2	100	0.0	0.53	69	8.509	0.997	0.993
	1	1	0.2	0.53	3	7.531	0.996	0.112
	2	1	0.2	0.53	16	7.045	0.996	0.998
	1	100	0.2	0.53	13	10.087	0.996	0.653
	2	100	0.2	0.53	80	9.964	0.996	0.999

Here, $\pi ^*$ represents the optimal proportion allocated to LTG

## Conclusions

Discrete competing-risks analysis has gained substantial interest in the research community and is now commonly employed in various fields, including biostatistics, econometrics, and educational research (Schmid and Berger 2021). However, design issues for such models have not been addressed. We believe this is the first paper that focuses on constructing optimal designs for collecting discrete-time survival data efficiently in longitudinal trials with competing risks.

Our methodological approach to identifying the desired optimal designs can be summarized as follows. We first utilize the framework of the generalized linear model to derive the information matrix for the discrete-time survival model with competing risks. To ensure efficient parameter estimation while considering cost constraints, we propose a general $D_{\textbf{A}}$-optimal design criterion, which is a function of the cost-normalized information matrix. We then assume that the underlying event processes can be modeled using a parametric competing risks model. Through simulations of various real-life scenarios, we find that the optimal designs depend on parameters of the Weibull distributions, cost ratios, attrition rates, design objectives, and models. Therefore, experiment designers should carefully consider these factors when designing trials. Our study suggests that when competing risks exist, equal allocation of patients to different groups is generally a favorable choice, unless both experimental groups exhibit low hazard rates throughout the trial duration. Additionally, our methods can be employed to find optimal designs even when the baseline risk function is not Weibull and the baseline coefficients are fitted using a polynomial function.

Our primary focus was on trials involving two randomized treatment groups. In Web Appendix B, we present corresponding simulation results for a scenario with three randomized groups: one placebo group and two treatment groups. The code for deriving these optimal designs is also included in the Supplementary Material accompanying this paper. Notably, the optimal designs for estimating treatment efficiencies in the three-group scenario do not necessarily exhibit the same properties as those for the two-group scenario, indicating that each scenario must be studied independently. As theoretically established in Remark 2, the proposed methodology can be extended to create optimal designs specifically tailored for the efficient estimation of the CIF targeting a particular failure type. A thorough investigation of these theoretical extensions, along with their empirical performance, will be presented in future work. While our numerical investigations confirmed the positive definiteness of the FIM for all scenarios in this study, a general analytical proof, akin to that pursued in Bu et al. (2020), remains an open problem. Establishing such theoretical guarantees for discrete-time survival models with competing risks constitutes a worthwhile direction for future methodological research.

A key consideration in this work is the choice of the cause-specific hazards framework for modeling discrete-time competing risks data. This approach was selected primarily for its methodological continuity with prior work on single-event optimal designs (Moerbeek and Wong 2015; Zhou et al. 2021), which facilitated a tractable derivation of the FIM, the cornerstone of our optimal design calculations. Furthermore, the cause-specific model remains a widely adopted and computationally practical choice for the analysis of discrete-time survival data (Schmid and Berger 2021).

However, we acknowledge that when the primary scientific interest lies in the CIF itself, direct regression models for the CIF can offer a more parsimonious and interpretable summary of covariate effects, as pointed by the reviewer. Two prominent strategies for direct CIF modeling are particularly relevant: the discrete subdistribution hazard model, an extension of the Fine-Gray model to discrete time (Schmid and Berger 2021), and direct transformation models that link the CIF to covariates (Lee 2017; Gerds et al. 2012). These approaches allow researchers to directly quantify how predictors influence the probability of a specific event occurring over time, without the need to model all cause-specific hazards.

Despite their inferential advantages, the adoption of these direct models in optimal design faces a major barrier: the formidable analytical complexity of deriving a closed-form FIM. Consequently, while our work establishes a foundational framework using the cause-specific model, a critical future direction is to develop novel computational or semi-parametric methods to enable optimal design for these powerful direct CIF models.

Furthermore, our framework naturally extends to the derivation of approximately optimal designs for continuous-time survival models with competing risks. This extension addresses an important gap in the optimal design literature, although similar concepts have been investigated in parallel research domains (Suresh et al. 2022; Kvamme and Borgan 2021).

## Supplementary Information

Below is the link to the electronic supplementary material.

Supplementary file 1 (pdf 431 KB)

Supplementary file 2 (R 15 KB)

Supplementary file 3 (R 10 KB)

## Derivation of the Fisher information matrix

According to the log-likelihood function (7), the score function is

$$\begin{aligned} \frac{\partial l({\boldsymbol{\theta }})}{\partial {\boldsymbol{\theta }}}= & \sum _{i=1}^N \sum _{t=1}^{t_{i}}\left( \frac{\partial {\boldsymbol{\lambda }}_{it}}{\partial {\boldsymbol{\theta }}^\mathrm{{T}}}\right) ^\mathrm{{T}}\operatorname {diag}\left( {\boldsymbol{\lambda }}_{it}\right) ^{-1} \textbf{Y}_{it},\\ \frac{\partial l({\boldsymbol{\theta }})}{\partial {\boldsymbol{\theta }}^\mathrm{{T}}}= & \left( \frac{\partial l}{\partial {\boldsymbol{\theta }}}\right) ^\mathrm{{T}}=\sum _{i=1}^N \sum _{t=1}^{t_{i}}\textbf{Y}_{it}^\mathrm{{T}}\operatorname {diag}\left( {\boldsymbol{\lambda }}_{it}\right) ^{-1} \frac{\partial {\boldsymbol{\lambda }}_{it}}{\partial {\boldsymbol{\theta }}^\mathrm{{T}}}. \end{aligned}$$

Let ${\boldsymbol{\eta }}_{it}^\mathrm{{T}}=(\eta _1(t|\textbf{x}_i),\ldots ,\eta _J(t|\textbf{x}_i),\eta _{J+1}(t|\textbf{x}_i))$. Using the formula of matrix differentiation, we have

$$\begin{aligned} \frac{\partial {\boldsymbol{\lambda }}_{it}}{\partial {\boldsymbol{\theta }}^\mathrm{{T}}}= & \frac{\partial {\boldsymbol{\lambda }}_{it}}{\partial {\boldsymbol{\eta }}_{it}^\mathrm{{T}}} \cdot \frac{\partial {\boldsymbol{\eta }}_{it}}{\partial {\boldsymbol{\theta }}^\mathrm{{T}}} \\= & \left( \frac{\partial {\boldsymbol{\eta }}_{it}}{\partial {\boldsymbol{\lambda }}_{it}^\mathrm{{T}}}\right) ^{-1} \cdot \textbf{X}_{it} \\= & \left( \frac{\partial \left[ \textbf{C}^\mathrm{{T}}\log \left( \textbf{L} {\boldsymbol{\lambda }}_{it}\right) \right] }{\partial \left[ \log \left( \textbf{L} {\boldsymbol{\lambda }}_{it}\right) \right] ^\mathrm{{T}}} \cdot \frac{\partial \left[ \log \left( \textbf{L}{\boldsymbol{\lambda }}_{it}\right) \right] }{\partial \left[ \textbf{L} {\boldsymbol{\lambda }}_{it}\right] ^\mathrm{{T}}} \cdot \frac{\partial \left[ \textbf{L} {\boldsymbol{\lambda }}_{it}\right] }{\partial {\boldsymbol{\lambda }}_{it}^\mathrm{{T}}}\right) ^{-1} \cdot \textbf{X}_{it}\\= & \left( \textbf{C}^\mathrm{{T}}\left[ \operatorname {diag}\left( \textbf{L} {\boldsymbol{\lambda }}_{it}\right) \right] ^{-1} \textbf{L}\right) ^{-1} \textbf{X}_{it}. \end{aligned}$$

It follows that the FIM is

$$\begin{aligned} \textbf{M}= & E\left( \frac{\partial l({\boldsymbol{\theta }})}{\partial {\boldsymbol{\theta }}}\cdot \frac{\partial l({\boldsymbol{\theta }})}{\partial {\boldsymbol{\theta }}^\mathrm{{T}}} \right) \nonumber \\= & E\left( \sum _{{i_1}=1}^N \sum _{{t_1}=1}^{t_{i_1}}\left( \frac{\partial {\boldsymbol{\lambda }}_{i_1t_1}}{\partial {\boldsymbol{\theta }}^\mathrm{{T}}}\right) ^\mathrm{{T}}\operatorname {diag}\left( {\boldsymbol{\lambda }}_{i_1t_1}\right) ^{-1} \textbf{Y}_{i_1t_1}\cdot \sum _{i_2=1}^N \sum _{t_2=1}^{t_{i_2}}\textbf{Y}_{i_2t_2}^\mathrm{{T}}\operatorname {diag}\left( {\boldsymbol{\lambda }}_{i_2t_2}\right) ^{-1} \frac{\partial {\boldsymbol{\lambda }}_{i_2t_2}}{\partial {\boldsymbol{\theta }}^\mathrm{{T}}} \right) \nonumber \\= & \sum _{{i_1}=1}^N \sum _{{t_1}=1}^{t_{i_1}}\sum _{i_2=1}^N \sum _{t_2=1}^{t_{i_2}} \left( \frac{\partial {\boldsymbol{\lambda }}_{i_1t_1}}{\partial {\boldsymbol{\theta }}^\mathrm{{T}}}\right) ^\mathrm{{T}}\operatorname {diag}\left( {\boldsymbol{\lambda }}_{i_1t_1}\right) ^{-1} E\left( \textbf{Y}_{i_1t_1} \textbf{Y}_{i_2t_2}^\mathrm{{T}}\right) \operatorname {diag}\left( {\boldsymbol{\lambda }}_{i_2t_2}\right) ^{-1} \frac{\partial {\boldsymbol{\lambda }}_{i_2t_2}}{\partial {\boldsymbol{\theta }}^\mathrm{{T}}}, \end{aligned}$$

where for $$ i_1=i_2=i,t_1=t_2=t,$$

$$\begin{aligned} E\left( \textbf{Y}_{it} \textbf{Y}_{it}^\mathrm{{T}}\right) =\operatorname {diag}({\boldsymbol{\lambda }}_{it}), \end{aligned}$$

and for $i_1\ne i_2$ or $t_1\ne t_2$,

$$\begin{aligned} E\left( \textbf{Y}_{i_1t_1} \textbf{Y}_{i_2t_2}^\mathrm{{T}}\right) ={\boldsymbol{\lambda }}_{i_1t_1}{\boldsymbol{\lambda }}_{i_2t_2}^\mathrm{{T}}. \end{aligned}$$

By Lemma S.5 of Bu et al. (2020), the sought FIM is

$$\begin{aligned} \textbf{M}= & \sum _{{i}=1}^N \sum _{{t}=1}^{t_{i}}\left( \frac{\partial {\boldsymbol{\lambda }}_{it}}{\partial {\boldsymbol{\theta }}^\mathrm{{T}}}\right) ^\mathrm{{T}}\left[ \operatorname {diag}({\boldsymbol{\lambda }}_{it})\right] ^{-1}\frac{\partial {\boldsymbol{\lambda }}_{it}}{\partial {\boldsymbol{\theta }}^\mathrm{{T}}}, \end{aligned}$$

where $$\frac{\partial {\boldsymbol{\lambda }}_{it}}{\partial {\boldsymbol{\theta }}^\mathrm{{T}}}=\left( \textbf{C}^\mathrm{{T}}\textbf{D}_{it}^{-1}\textbf{L}\right) ^{-1}\textbf{X}_{it}$$ and $$\textbf{D}_{it}=\operatorname {diag}(\textbf{L}{\boldsymbol{\lambda }}_{it})$$.
