# Supplementary material for: Optimal designs for discrete-time survival models with competing risks
Source: Lifetime Data Anal. 2026 Feb 28;32(2):17. doi: 10.1007/s10985-026-09695-0 (PMC12950086; doi:10.1007/s10985-026-09695-0)
Supplement: Supplementary file 1 — (pdf 431 KB) [file 10985_2026_9695_MOESM1_ESM.pdf]

Noname manuscript No.
(will be inserted by the editor)

Optimal designs for discrete-time survival models with competing risks

Supplementary Materials

The supplementary materials has 2 sections. In Section 1, we display tables of designs under various setups, including graphs of hazard functions for different time periods (Web Appendix A). In Section 2, we present additional simulation results for optimal designs for comparing two treatment groups with a placebo group (Web Appendix B).

Address(es) of author(s) should be given

Web Appendix A. Sensitivity analysis to various parameter settings

Table S1 D_s-optimal designs $\zeta^* = \{q^*\}$ for model (17) under different values of w_r, τ_r, and γ_{r1} $(r = 1, 2)$, with $\kappa = 0.5$, $f = 100$, $\rho = 0$, and equal group allocation.

τ_1	τ_2	w_1	w_2	$(\gamma_{11}, \gamma_{21})$			
				(-2.5,-2.5)	(-2.5,2.5)	(2.5,-2.5)	(2.5,2.5)
1/3	1/3	0.3	0.3	12	12	12	12
1/3	1/3	0.3	0.5	12	12	12	12
1/3	1/3	0.5	0.3	12	12	12	12
1/3	1/3	0.5	0.5	12	12	12	12
1/3	3	0.3	0.3	12	12	12	12
1/3	3	0.3	0.5	12	12	12	12
1/3	3	0.5	0.3	12	12	12	12
1/3	3	0.5	0.5	12	12	12	12
3	3	0.3	0.3	12	12	12	12
3	3	0.3	0.5	12	12	12	12
3	3	0.5	0.3	12	12	12	12
3	3	0.5	0.5	12	12	12	12

Table S2 D_s-optimal designs $\zeta^* = \{q^*\}$ for model (17) under different values of w_r, τ_r, and γ_{r1} $(r = 1, 2)$, with $\kappa = 0.5$, $f = 1$, $\rho = 0.3$, and equal group allocation.

τ_1	τ_2	w_1	w_2	$(\gamma_{11}, \gamma_{21})$			
				(-2.5,-2.5)	(-2.5,2.5)	(2.5,-2.5)	(2.5,2.5)
1/3	1/3	0.3	0.3	1	1	1	1
1/3	1/3	0.3	0.5	1	1	1	1
1/3	1/3	0.5	0.3	1	1	1	1
1/3	1/3	0.5	0.5	1	1	1	1
1/3	3	0.3	0.3	12	12	12	12
1/3	3	0.3	0.5	12	12	12	12
1/3	3	0.5	0.3	12	12	12	12
1/3	3	0.5	0.5	12	12	12	12
3	3	0.3	0.3	12	12	12	12
3	3	0.3	0.5	12	12	12	12
3	3	0.5	0.3	12	12	12	12
3	3	0.5	0.5	12	12	12	12

Table S3 D_s-optimal designs $\zeta^* = \{q^*\}$ for model (17) under varying parameters $w_r, \tau_r, \gamma_{r1}, r = 1, 2$ with $\kappa = 0.3$, $f = 1$, $\rho = 0$, and equal group allocation.

τ_1	τ_2	w_1	w_2	$(\gamma_{11}, \gamma_{21})$			
				(-2.5,-2.5)	(-2.5,2.5)	(2.5,-2.5)	(2.5,2.5)
1/3	1/3	0.3	0.3	1	1	1	1
1/3	1/3	0.3	0.5	1	1	1	1
1/3	1/3	0.5	0.3	1	1	1	1
1/3	1/3	0.5	0.5	1	1	1	1
1/3	3	0.3	0.3	12	12	12	12
1/3	3	0.3	0.5	12	11	12	10
1/3	3	0.5	0.3	12	12	12	11
1/3	3	0.5	0.5	12	12	12	9
3	3	0.3	0.3	12	12	12	12
3	3	0.3	0.5	12	12	12	12
3	3	0.5	0.3	12	12	12	12
3	3	0.5	0.5	12	11	12	9

Table S4 D_s-optimal designs $\zeta^* = \{q^*\}$ for model (17) under varying parameters $w_r, \tau_r, \gamma_{r1}, r = 1, 2$ with $\kappa = 0.7$, $f = 1$, $\rho = 0$, and equal group allocation.

τ_1	τ_2	w_1	w_2	$(\gamma_{11}, \gamma_{21})$			
				(-2.5,-2.5)	(-2.5,2.5)	(2.5,-2.5)	(2.5,2.5)
1/3	1/3	0.3	0.3	1	1	1	1
1/3	1/3	0.3	0.5	1	1	1	1
1/3	1/3	0.5	0.3	1	1	1	1
1/3	1/3	0.5	0.5	1	1	1	1
1/3	3	0.3	0.3	12	12	12	12
1/3	3	0.3	0.5	12	12	12	11
1/3	3	0.5	0.3	12	12	12	12
1/3	3	0.5	0.5	12	12	12	10
3	3	0.3	0.3	12	12	12	12
3	3	0.3	0.5	12	12	12	12
3	3	0.5	0.3	12	12	12	12
3	3	0.5	0.5	12	11	12	10

Table S5 D_s-optimal designs $\zeta^* = \{\pi^*, q^*\}$ for different $w_r, \tau_r, r = 1, 2, \gamma_{11}, \gamma_{21}$ when the cost ratio is $f = 100$, the proportion parameter $\kappa = 0.5$, and the attrition rate is $\rho = 0$. Here, π^* denotes the optimal proportion allocated to the experimental group.

				$(\gamma_{11}, \gamma_{21})$											
τ_1	τ_2	w_1	w_2	(-2.5,-2.5)			(-2.5,2.5)			(2.5,-2.5)			(2.5,2.5)		
				π^*	q^*	RE	π^*	q^*	RE	π^*	q^*	RE	π^*	q^*	RE
1/3	1/3	0.3	0.3	0.76	12	0.79	0.56	12	0.99	0.56	12	0.99	0.40	12	0.96
1/3	1/3	0.3	0.5	0.75	12	0.80	0.60	12	0.97	0.56	12	0.99	0.44	12	0.99
1/3	1/3	0.5	0.3	0.75	12	0.80	0.56	12	0.99	0.60	12	0.97	0.44	12	0.99
1/3	1/3	0.5	0.5	0.74	12	0.82	0.60	12	0.97	0.60	12	0.97	0.47	12	1.00
3	1/3	0.3	0.3	0.76	12	0.79	0.57	12	0.99	0.54	12	1.00	0.41	12	0.97
3	1/3	0.3	0.5	0.75	12	0.80	0.61	12	0.96	0.53	12	1.00	0.48	12	1.00
3	1/3	0.5	0.3	0.76	12	0.79	0.57	12	0.99	0.57	12	0.99	0.42	12	0.98
3	1/3	0.5	0.5	0.75	12	0.81	0.61	12	0.96	0.56	12	0.99	0.48	12	1.00
3	3	0.3	0.3	0.76	12	0.79	0.55	12	0.99	0.55	12	0.99	0.38	12	0.95
3	3	0.3	0.5	0.75	12	0.80	0.59	12	0.98	0.55	12	0.99	0.40	12	0.97
3	3	0.5	0.3	0.75	12	0.80	0.55	12	0.99	0.59	12	0.98	0.40	12	0.97
3	3	0.5	0.5	0.75	12	0.81	0.59	12	0.98	0.59	12	0.98	0.41	12	0.98

Table S6 D_s-optimal designs $\zeta^* = \{\pi^*, q^*\}$ for different $w_r, \tau_r, r = 1, 2, \gamma_{11}, \gamma_{21}$ when the cost ratio is $f = 1$, the proportion parameter $\kappa = 0.5$, and the attrition rate is $\rho = 0.3$. Here, π^* denotes the optimal proportion allocated to the experimental group.

				$(\gamma_{11}, \gamma_{21})$											
τ_1	τ_2	w_1	w_2	(-2.5,-2.5)			(-2.5,2.5)			(2.5,-2.5)			(2.5,2.5)		
				π^*	q^*	RE	π^*	q^*	RE	π^*	q^*	RE	π^*	q^*	RE
1/3	1/3	0.3	0.3	0.76	1	0.79	0.56	1	0.99	0.56	1	0.99	0.38	1	0.94
1/3	1/3	0.3	0.5	0.75	1	0.80	0.60	1	0.97	0.55	1	0.99	0.42	1	0.97
1/3	1/3	0.5	0.3	0.75	1	0.80	0.55	1	0.99	0.60	1	0.97	0.42	1	0.97
1/3	1/3	0.5	0.5	0.74	1	0.81	0.60	1	0.97	0.60	1	0.97	0.45	1	0.99
3	1/3	0.3	0.3	0.76	12	0.78	0.57	12	0.99	0.51	12	1.00	0.36	12	0.93
3	1/3	0.3	0.5	0.75	12	0.80	0.61	12	0.97	0.51	12	1.00	0.43	12	0.98
3	1/3	0.5	0.3	0.76	12	0.79	0.57	12	0.99	0.53	12	1.00	0.37	12	0.94
3	1/3	0.5	0.5	0.75	12	0.80	0.61	12	0.97	0.52	12	1.00	0.43	12	0.98
3	3	0.3	0.3	0.77	12	0.78	0.53	12	1.00	0.53	12	1.00	0.30	12	0.87
3	3	0.3	0.5	0.76	12	0.78	0.54	12	0.99	0.52	12	1.00	0.31	12	0.89
3	3	0.5	0.3	0.76	12	0.78	0.52	12	1.00	0.54	12	0.99	0.31	12	0.89
3	3	0.5	0.5	0.76	12	0.79	0.54	12	1.00	0.54	12	1.00	0.32	12	0.90

Table S7 D_s-optimal designs $\zeta^* = \{\pi^*, q^*\}$ for model (17) under varying parameters w_r, τ_r, γ_{r1}, $r = 1, 2$ with a cost ratio of $f = 1$, a proportion parameter of $\kappa = 0.3$, and an attrition rate of $\rho = 0$. Here π^* denotes the optimal proportion allocated to the experimental group.

				$(\gamma_{11}, \gamma_{21})$											
τ_1	τ_2	w_1	w_2	(-2.5,-2.5)			(-2.5,2.5)			(2.5,-2.5)			(2.5,2.5)		
				π^*	q^*	RE	π^*	q^*	RE	π^*	q^*	RE	π^*	q^*	RE
1/3	1/3	0.3	0.3	0.76	1	0.79	0.58	1	0.98	0.53	1	1.00	0.40	1	0.94
1/3	1/3	0.3	0.5	0.74	1	0.81	0.64	1	0.95	0.53	1	1.00	0.45	1	0.98
1/3	1/3	0.5	0.3	0.75	1	0.80	0.58	1	0.98	0.56	1	0.99	0.42	1	0.96
1/3	1/3	0.5	0.5	0.74	1	0.81	0.64	1	0.95	0.56	1	0.99	0.47	1	0.99
3	1/3	0.3	0.3	0.76	12	0.79	0.59	12	0.98	0.51	12	1.00	0.44	12	0.98
3	1/3	0.3	0.5	0.74	12	0.81	0.65	12	0.93	0.50	12	1.00	0.53	12	1.00
3	1/3	0.5	0.3	0.75	12	0.79	0.59	12	0.98	0.53	12	1.00	0.45	11	0.99
3	1/3	0.5	0.5	0.74	12	0.81	0.65	12	0.93	0.52	12	1.00	0.53	10	1.00
3	3	0.3	0.3	0.76	12	0.79	0.58	12	0.98	0.53	12	1.00	0.38	12	0.95
3	3	0.3	0.5	0.75	12	0.80	0.62	12	0.96	0.52	12	1.00	0.41	11	0.97
3	3	0.5	0.3	0.76	12	0.79	0.58	12	0.98	0.55	12	0.99	0.39	12	0.96
3	3	0.5	0.5	0.75	12	0.81	0.62	12	0.97	0.55	12	0.99	0.41	11	0.97

Table S8 D_s-optimal designs $\zeta^* = \{\pi^*, q^*\}$ for model (17) under varying parameters w_r, τ_r, γ_{r1}, $r = 1, 2$ with a cost ratio of $f = 1$, a proportion parameter of $\kappa = 0.7$, and an attrition rate of $\rho = 0$. Here π^* denotes the optimal proportion allocated to the experimental group.

				$(\gamma_{11}, \gamma_{21})$											
τ_1	τ_2	w_1	w_2	(-2.5,-2.5)			(-2.5,2.5)			(2.5,-2.5)			(2.5,2.5)		
				π^*	q^*	RE	π^*	q^*	RE	π^*	q^*	RE	π^*	q^*	RE
1/3	1/3	0.3	0.3	0.76	1	0.79	0.53	1	1.00	0.58	1	0.98	0.40	1	0.94
1/3	1/3	0.3	0.5	0.75	1	0.80	0.56	1	0.99	0.58	1	0.98	0.42	1	0.96
1/3	1/3	0.5	0.3	0.74	1	0.81	0.53	1	1.00	0.64	1	0.95	0.45	1	0.98
1/3	1/3	0.5	0.5	0.74	1	0.81	0.56	1	0.99	0.64	1	0.95	0.47	1	0.99
3	1/3	0.3	0.3	0.76	12	0.78	0.54	12	1.00	0.56	12	0.99	0.37	11	0.94
3	1/3	0.3	0.5	0.76	12	0.79	0.57	12	0.99	0.56	12	0.99	0.42	11	0.97
3	1/3	0.5	0.3	0.76	12	0.79	0.54	12	1.00	0.60	11	0.98	0.39	9	0.95
3	1/3	0.5	0.5	0.75	12	0.80	0.57	12	0.99	0.59	11	0.98	0.43	9	0.97
3	3	0.3	0.3	0.76	12	0.79	0.53	12	1.00	0.58	12	0.98	0.38	12	0.95
3	3	0.3	0.5	0.76	12	0.79	0.55	12	0.99	0.58	12	0.98	0.39	12	0.96
3	3	0.5	0.3	0.75	12	0.80	0.52	12	1.00	0.62	12	0.96	0.41	11	0.97
3	3	0.5	0.5	0.75	12	0.81	0.55	12	0.99	0.62	12	0.97	0.41	11	0.97

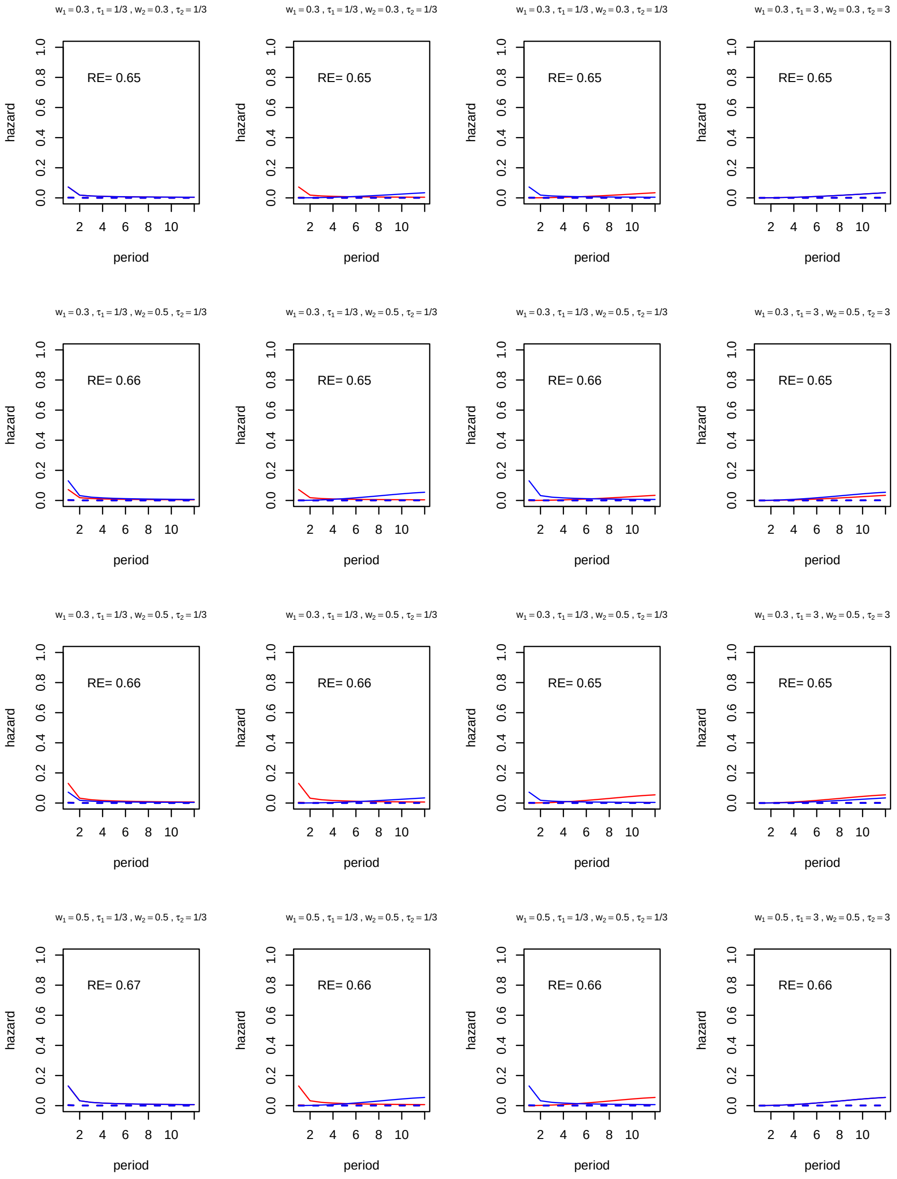

Fig. S1 Hazard functions of different time periods for different $w_r, \tau_r, r = 1, 2$ and the relative efficiencies of the the optimal designs with equal group sizes when the treatment effects are $\gamma_{11} = \gamma_{21} = -4$. Different colors are used to distinguish hazard functions for different risks, and hazard functions for treatment groups are shown as dotted lines.

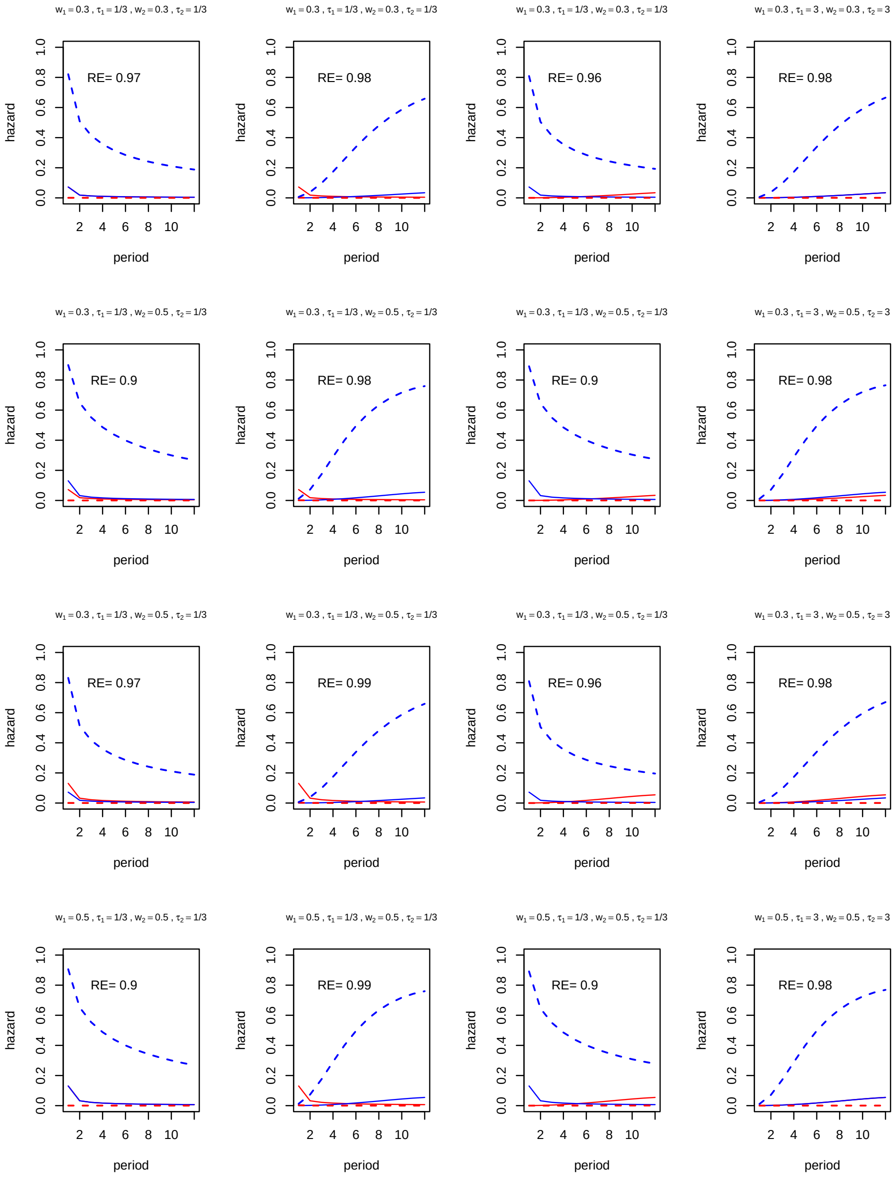

Fig. S2 Hazard functions of time periods for different $w_r, \tau_r, r = 1, 2$ and the relative efficiencies of the the optimal designs with equal group sizes when the treatment effects are $\gamma_{11} = -4$ and $\gamma_{21} = 4$. Different colors are used to distinguish hazard functions for different risks, and hazard functions for treatment groups are shown as dotted lines.

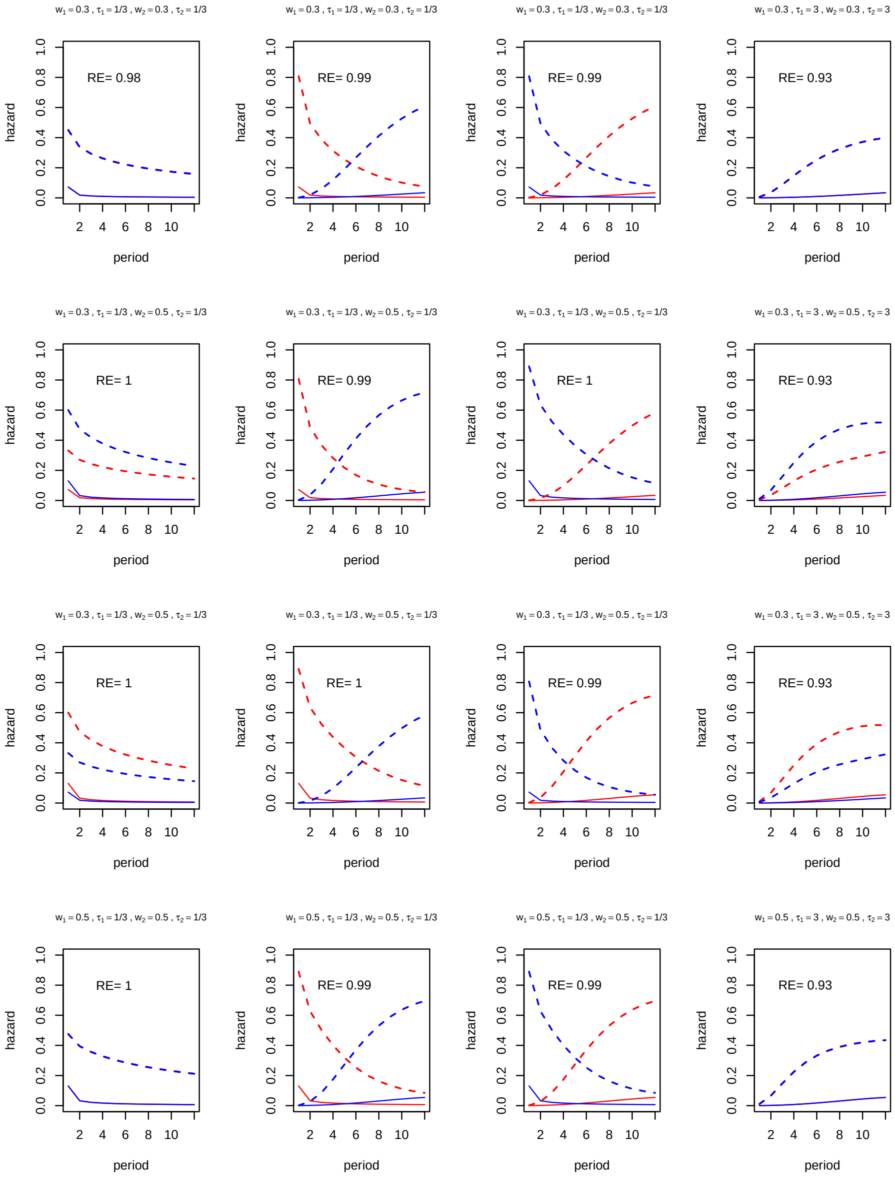

Fig. S3 Hazard functions of time periods for different $w_r, \tau_r, r = 1, 2$ and the relative efficiencies of the the optimal designs with equal group sizes when the treatment effects are $\gamma_{11} = \gamma_{2,1} = 4$. Different colors are used to distinguish hazard functions for different risks and, hazard functions for treatment groups are shown as dotted lines.

Table S9 Locally D_{s_1}-optimal designs $\zeta^* = \{\pi^*, q^*\}$ and the efficiencies of the optimal designs with equal group sizes for various τ_r,$\gamma_{r2}, r = 1, 2$and $f_i, i = 1, 2$ when $\gamma_{11} = \gamma_{21} = 0.3$, $w_r = 0.3$, $r = 1, 2$, $\kappa = 0.5$ and $\rho = 0$. Here, π^* denotes the optimal proportion allocated to the experimental group.

f_1	f_2	τ_1	τ_2	γ_{12}	$\gamma_{22} = -0.5$			$\gamma_{22} = 0$			$\gamma_{22} = 0.5$		
					π^*	q^*	RE	π^*	q^*	RE	π^*	q^*	RE
1	1	1/3	1/3	-0.5	0.55	5	0.99	0.52	9	1.00	0.46	12	1.00
				0.5	0.46	12	1.00	0.43	12	0.99	0.38	12	0.95
		1/3	3	-0.5	0.65	10	0.93	0.52	12	1.00	0.45	10	0.99
				0.5	0.52	12	1.00	0.46	12	0.99	0.37	10	0.95
		3	3	-0.5	0.73	12	0.84	0.58	12	0.98	0.52	11	1.00
				0.5	0.52	11	1.00	0.46	11	0.99	0.36	10	0.94
1	100	1/3	1/3	-0.5	0.59	12	0.97	0.53	12	1.00	0.46	12	1.00
				0.5	0.46	12	1.00	0.43	12	0.99	0.38	12	0.95
		1/3	3	-0.5	0.66	12	0.92	0.52	12	1.00	0.47	11	1.00
				0.5	0.52	12	1.00	0.46	12	0.99	0.38	11	0.95
		3	3	-0.5	0.73	12	0.84	0.58	12	0.98	0.52	11	1.00
				0.5	0.52	11	1.00	0.46	11	0.99	0.37	11	0.94
100	100	1/3	1/3	-0.5	0.59	12	0.97	0.53	12	1.00	0.46	12	1.00
				0.5	0.46	12	1.00	0.43	12	0.99	0.38	12	0.95
		1/3	3	-0.5	0.66	12	0.92	0.52	12	1.00	0.47	12	1.00
				0.5	0.52	12	1.00	0.46	12	0.99	0.38	12	0.95
		3	3	-0.5	0.73	12	0.84	0.58	12	0.98	0.52	12	1.00
				0.5	0.52	12	1.00	0.46	12	0.99	0.37	11	0.95

Table S10 Locally D_{s_2}-optimal designs $\zeta^* = \{\pi^*, q^*\}$ and the relative efficiencies of the optimal designs with equal group sizes for various τ_r, $\gamma_{r2}, r = 1, 2$ and $f_i, i = 1, 2$ when $\gamma_{11} = \gamma_{21} = 0.3$ and $w_r = 0.3$, $r = 1, 2$, $\kappa = 0.5$ and $\rho = 0$. Here, π^* denotes the optimal proportion allocated to the experimental group.

f_1	f_2	τ_1	τ_2	γ_{12}	$\gamma_{22} = -0.5$			$\gamma_{22} = 0$			$\gamma_{22} = 0.5$		
					π^*	q^*	RE	π^*	q^*	RE	π^*	q^*	RE
1	1	1/3	1/3	-0.5	0.55	5	0.99	0.56	5	0.99	0.56	5	0.99
				0.5	0.34	12	0.91	0.34	12	0.91	0.38	12	0.95
		1/3	3	-0.5	0.55	5	0.99	0.55	5	0.99	0.55	5	0.99
				0.5	0.33	12	0.91	0.33	12	0.91	0.38	10	0.95
		3	3	-0.5	0.73	12	0.84	0.73	12	0.84	0.69	8	0.89
				0.5	0.34	11	0.92	0.34	11	0.93	0.36	10	0.94
1	100	1/3	1/3	-0.5	0.59	12	0.97	0.59	12	0.97	0.58	9	0.98
				0.5	0.34	12	0.91	0.34	12	0.91	0.38	12	0.95
		1/3	3	-0.5	0.59	11	0.97	0.59	11	0.97	0.57	8	0.98
				0.5	0.33	12	0.91	0.33	12	0.91	0.39	11	0.96
		3	3	-0.5	0.73	12	0.84	0.73	12	0.84	0.70	10	0.88
				0.5	0.34	12	0.93	0.35	12	0.93	0.37	11	0.95
100	100	1/3	1/3	-0.5	0.59	12	0.97	0.59	12	0.97	0.58	10	0.98
				0.5	0.34	12	0.91	0.34	12	0.91	0.38	12	0.95
		1/3	3	-0.5	0.59	12	0.97	0.59	12	0.97	0.58	9	0.98
				0.5	0.33	12	0.91	0.33	12	0.91	0.39	12	0.96
		3	3	-0.5	0.73	12	0.84	0.73	12	0.84	0.70	10	0.88
				0.5	0.34	12	0.93	0.35	12	0.93	0.37	11	0.95

Table S11 D-optimal designs $\zeta^* = \{\pi^*, q^*\}$ and the relative efficiencies of the optimal designs with equal group sizes for various τ_r, $\gamma_{r2}, r = 1, 2$ and $f_i, i = 1, 2$ when $\gamma_{11} = \gamma_{21} = 0.3$, $w_r = 0.3$, $r = 1, 2$, $\kappa = 0.3$ and $\rho = 0$. Here, π^* denotes the optimal proportion allocated to the experimental group.

f_1	f_2	τ_1	τ_2	γ_{12}	$\gamma_{22} = -0.5$			$\gamma_{22} = 0$			$\gamma_{22} = 0.5$		
					π^*	q^*	RE	π^*	q^*	RE	π^*	q^*	RE
1	1	1/3	1/3	-0.5	0.50	2	1.00	0.50	2	1.00	0.50	2	1.00
				0.5	0.50	2	1.00	0.50	2	1.00	0.50	2	1.00
		1/3	3	-0.5	0.49	3	1.00	0.48	4	1.00	0.50	5	1.00
				0.5	0.55	5	1.00	0.72	10	0.94	0.75	8	0.91
		3	3	-0.5	0.18	12	0.85	0.31	12	0.97	0.36	12	0.99
				0.5	0.48	11	1.00	0.68	10	0.96	0.76	9	0.91
1	100	1/3	1/3	-0.5	0.50	2	1.00	0.49	3	1.00	0.51	3	1.00
				0.5	0.51	3	1.00	0.56	4	1.00	0.73	7	0.95
		1/3	3	-0.5	0.23	9	0.95	0.33	10	0.97	0.48	8	1.00
				0.5	0.52	12	1.00	0.73	12	0.95	0.75	8	0.91
		3	3	-0.5	0.18	12	0.85	0.31	12	0.97	0.36	12	0.97
				0.5	0.44	12	1.00	0.65	11	0.98	0.76	9	0.92
100	100	1/3	1/3	-0.5	0.50	2	1.00	0.49	3	1.00	0.51	3	1.00
				0.5	0.51	3	1.00	0.56	4	0.99	0.73	7	0.94
		1/3	3	-0.5	0.19	11	0.92	0.31	11	0.97	0.48	8	1.00
				0.5	0.52	12	1.00	0.73	12	0.95	0.75	8	0.91
		3	3	-0.5	0.18	12	0.85	0.31	12	0.97	0.36	12	0.97
				0.5	0.44	12	1.00	0.65	11	0.98	0.76	9	0.92

Table S12 D-optimal designs $\zeta^* = \{\pi^*, q^*\}$ and the relative efficiencies of the optimal designs with equal group sizes for various τ_r, $\gamma_{r2}, r = 1, 2$ and $f_i, i = 1, 2$ when $\gamma_{11} = \gamma_{21} = 0.3$, $w_r = 0.3$, $r = 1, 2$, $\kappa = 0.7$ and $\rho = 0$. Here, π^* denotes the optimal proportion allocated to the experimental group.

f_1	f_2	τ_1	τ_2	γ_{12}	$\gamma_{22} = -0.5$			$\gamma_{22} = 0$			$\gamma_{22} = 0.5$		
					π^*	q^*	RE	π^*	q^*	RE	π^*	q^*	RE
1	1	1/3	1/3	-0.5	0.50	2	1.00	0.50	2	1.00	0.50	2	1.00
				0.5	0.50	2	1.00	0.50	2	1.00	0.50	2	1.00
		1/3	3	-0.5	0.49	3	1.00	0.48	4	1.00	0.50	6	1.00
				0.5	0.53	5	1.00	0.65	7	0.97	0.75	8	0.92
		3	3	-0.5	0.18	12	0.85	0.32	12	0.97	0.48	11	1.00
				0.5	0.36	12	0.99	0.65	9	0.98	0.76	9	0.91
1	100	1/3	1/3	-0.5	0.50	2	1.00	0.50	3	1.00	0.51	3	1.00
				0.5	0.51	3	1.00	0.53	3	1.00	0.73	7	0.95
		1/3	3	-0.5	0.21	10	0.94	0.31	11	0.97	0.49	9	1.00
				0.5	0.50	10	1.00	0.67	10	0.97	0.77	9	0.90
		3	3	-0.5	0.18	12	0.85	0.32	12	0.97	0.44	12	1.00
				0.5	0.36	12	0.97	0.45	12	1.00	0.76	9	0.92
100	100	1/3	1/3	-0.5	0.50	2	1.00	0.50	3	1.00	0.51	3	1.00
				0.5	0.51	3	1.00	0.53	3	1.00	0.73	7	0.94
		1/3	3	-0.5	0.17	12	0.91	0.29	12	0.96	0.49	9	1.00
				0.5	0.50	10	1.00	0.67	10	0.97	0.77	9	0.90
		3	3	-0.5	0.18	12	0.85	0.32	12	0.97	0.44	12	1.00
				0.5	0.36	12	0.97	0.45	12	1.00	0.76	9	0.92

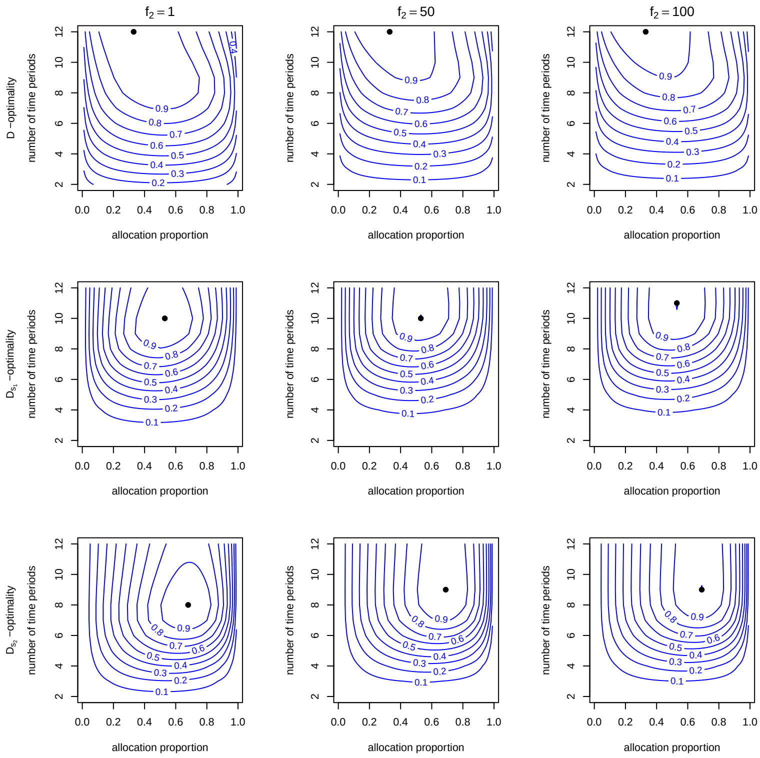

Fig. S4 Relative efficiencies as functions of q, the number of time periods, and π , the allocation proportion, with different cost ratios and design criteria, for the case $\gamma_{11} = 0.3, \gamma_{12} = -0.5, \gamma_{21} = 0.3, \gamma_{22} = 0.5$, $(w_1, \tau_1) = (0.3, 3), (w_2, \tau_2) = (0.5, 3)$ and $\rho = 0$.

Web Appendix B. Optimal designs for comparing two treatment groups with a placebo group

In this Section, we provide corresponding optimal designs for the DTSM with 3 groups and two competing risks, i.e. two treatment groups and a placebo group. The statistical model extends the one given in Equation (17) and is given by:

$$\eta_r(t|x) = \gamma_{0tr} + \gamma_{r1}x_1 + \gamma_{r2}x_2, r = 1, 2, \quad t = 1, \ldots, q. \tag{S1}$$

Here, x_1 and x_2 are treatment indicators corresponding to the treatment 1 and treatment 2, respectively. The group with $x_1 = 1, x_2 = 0$ focuses on comparing treatment 1 group with the placebo group. Similarly, the group with $x_1 = 0, x_2 = 1$ focuses on comparing treatment 2 group with the placebo group. The coefficients γ_{11} and γ_{21} in model (S1) represent the effects of the treatment 1 on the interested events 1 and 2, respectively. Similarly, the parameters γ_{12} and γ_{22} in model (S1) represent the effects of the treatment 2 on the interested events 1 and 2, respectively. For ease of presentation, we assume that the proportion of subjects allocated to the treatment i group is denoted by $\pi_{i+1}, i = 1, 2$. Then the proportion of subjects allocated to the placebo group is $\pi_1 = 1 - \pi_2 - \pi_3$.

The nominal values of the parameters in Weibull distribution are the same as those in Subsection 4.2. Furthermore, we assume that γ_{11} has values -2.5 or 2.5, γ_{21} has values -1.5 or 1.5, γ_{12} has values -1 or 1, and γ_{22} has values -0.5 or 0.5. As before, the design criteria are: locally D-optimality for estimating all model parameters $\boldsymbol{\theta}$ in (S1), and three types of locally D_s-optimality for estimating three subsets of $\boldsymbol{\theta}$: D_{s_1}-optimality for estimating the effects of the various treatments $\gamma_{rj}, r = 1, 2, j = 1, 2$, D_{s_2}-optimality for estimating the effects of treatment 1 $\gamma_{1j}, j = 1, 2$ and D_{s_3}-optimality for estimating the effects of treatment 2 $\gamma_{2j}, j = 1, 2$. These optimal designs minimize the volume of the confidence ellipsoids of the estimated parameters of interest and they can be found by first selecting an appropriate matrix $\mathbf{A}$ in the design criterion (15) of Subsection 3.3. The criterion is then minimized by choosing the optimal values of proportions in the 3 groups $\pi_1^*, \pi_2^*, \pi_3^*$, along with the optimal choice of the number of the time periods q^* within the design region $\mathcal{X} = \{\zeta | \sum_{i=1}^{3} \pi_i = 1, \pi_i \in \{0.01, 0.02, \ldots, 0.99\}, q \in \{2, 3, \ldots, 12\}\}$. These 4 quantities define the optimal design for the DSTM when there are 3 different groups and the optimal design is generically denoted by ζ^*.

Figure S5 presents the weight distributions of the locally D-optimal designs for fixed treatment effects $(\gamma_{11}, \gamma_{21}, \gamma_{12}, \gamma_{22}) = (2.5, 1.5, 1, 0.5)$ under varying Weibull distribution configurations. For comparative analysis, Figures S6-S8 illustrate the corresponding results for the locally D_{s_1}-, D_{s_2}-, and D_{s_3}-optimal designs, respectively, all evaluated using the second cost function (Eq. 13). Collectively, these figures reveal systematic differences in weight distribution patterns across optimal design criteria. The locally D-optimal designs demonstrate significantly greater sensitivity to variations in the number of test intervals, exhibiting the most pronounced fluctuations in weight allocation, whereas the locally D_{s_1}-optimal designs maintain remarkable stability under identical conditions. Notably, when the design prioritizes accurate estimation of a specific treatment effect, a consistent allocation pattern emerges: weights assigned to non-target treatments approach negligible values, while the control group consistently receives maximal allocation across most experimental configurations. These observed patterns highlight the funda-

mental trade-offs between estimation precision and design robustness inherent in different optimization approaches.

Our analysis reveals that the optimal design weights are significantly influenced by the parameters $\tau_r, r = 1, 2$, with larger τ_r values resulting in more pronounced weight disparities among groups under D_{s_i}-optimal criteria. Through comprehensive efficiency calculations, we find that equal allocation designs demonstrate strong performance for D-optimality and D_{s_1}-optimality cases, maintaining efficiencies above 90% across most scenarios. However, this pattern markedly differs for $D_{s_i}, i = 2, 3$-optimality, where equal allocation efficiencies frequently fall below 70%. Notably, when $\tau_r = 3, r = 1, 2,$, the equal allocation strategy proves particularly inefficient, suggesting the need for alternative weighting approaches in these parameter regimes.

Table S13 presents locally D-optimal designs $\zeta^* = \{\pi_1^*, \pi_2^*, \pi_3^*, q^*\}$ and the efficiencies of the locally optimal design with equal group sizes for different parameter settings, assuming $w_1 = 0.3$, $w_2 = 0.5$ and $\rho = 0$. Results for other combinations of $w_r, r = 1, 2$ are very similar, so we do not present them. The results suggest that the parameters τ_r, cost ratios f_i and cost functions significantly influence the optimal time periods q^*. With increasing $\tau_r, r = 1, 2$, the optimal values of q^* tend to either remain constant or increase. Additionally, as $\tau_r, r = 1, 2$ increases, the variability in optimal weights among different experimental groups also increases, resulting in a decrease in the efficiency of the design with equal group sizes. Similarly, as the cost ratios f_i increase, the optimal values of q^* tend to either remain constant or increase. Furthermore, numerical results indicate that the optimal value of q^* in the locally optimal design for cost function 2 is greater than or equal to its corresponding value for cost function 1.

For subsets estimation, Tables S14 - S16 present locally optimal designs $\zeta^* = \{\pi_1^*, \pi_2^*, \pi_3^*, q^*\}$ and the efficiencies of the locally optimal designs with equal group sizes for the D_{s_1}-, D_{s_2} and D_{s_3}-optimality, respectively. From these tables, we observe that the efficiencies of the optimal designs with equal group sizes depend on the design objectives. When the objective is to efficiently estimate all treatment effects (D_{s_1}-optimality), the efficiencies approach 1. However, when the focus is on a specific treatment effect (D_{s_2} or D_{s_3}-optimality), the efficiencies of the optimal designs with equal group sizes are relatively poor, and the weights assigned to the other treatment group approaches 0.

Table S13 D-optimal designs $\zeta^* = \{\pi_1^*, \pi_2^*, \pi_3^*, q^*\}$ and the relative efficiencies of the optimal designs with equal group sizes for various τ_r, and $f_i, i = 1, 2$ when $(\gamma_{11}, \gamma_{21}, \gamma_{12}, \gamma_{22}) = (2.5, 1.5, 1, 0.5)$, $w_1 = 0.3$, $w_2 = 0.5$ and $\kappa = 0.5$.

τ_1	τ_2	f_1	f_2	f_3	Cost function 1					Cost function 2				
					π_1^*	π_2^*	π_3^*	q^*	RE	π_1^*	π_2^*	π_3^*	q^*	RE
1/3	1/3	1	1	1	0.30	0.36	0.34	2	1.00	0.30	0.36	0.34	2	1.00
1/3	1/3	1	1	100	0.30	0.36	0.34	2	1.00	0.30	0.36	0.34	2	1.00
1/3	1/3	1	100	100	0.30	0.36	0.34	2	1.00	0.30	0.36	0.34	2	1.00
1/3	1/3	50	50	50	0.30	0.36	0.34	2	1.00	0.30	0.36	0.34	2	1.00
1/3	1/3	50	100	100	0.30	0.36	0.34	2	1.00	0.30	0.36	0.34	2	1.00
1/3	1/3	100	100	100	0.30	0.36	0.34	2	1.00	0.30	0.36	0.34	2	1.00
3	1/3	1	1	1	0.24	0.48	0.28	3	0.97	0.24	0.48	0.28	3	0.97
3	1/3	1	1	100	0.16	0.63	0.21	7	0.91	0.16	0.63	0.21	7	0.91
3	1/3	1	100	100	0.16	0.63	0.21	7	0.91	0.15	0.65	0.20	8	0.91
3	1/3	50	50	50	0.16	0.63	0.21	7	0.91	0.16	0.63	0.21	7	0.91
3	1/3	50	100	100	0.16	0.63	0.21	7	0.91	0.15	0.65	0.20	8	0.91
3	1/3	100	100	100	0.15	0.65	0.20	8	0.91	0.15	0.65	0.20	8	0.91
3	3	1	1	1	0.12	0.73	0.15	9	0.83	0.11	0.74	0.15	10	0.84
3	3	1	1	100	0.11	0.73	0.16	11	0.86	0.11	0.73	0.16	11	0.87
3	3	1	100	100	0.11	0.73	0.16	11	0.87	0.11	0.73	0.16	11	0.87
3	3	50	50	50	0.11	0.73	0.16	11	0.87	0.11	0.73	0.16	11	0.87
3	3	50	100	100	0.11	0.73	0.16	11	0.87	0.12	0.70	0.18	12	0.88
3	3	100	100	100	0.11	0.73	0.16	11	0.87	0.12	0.70	0.18	12	0.88

Table S14 D_{s_1}-optimal designs $\zeta^* = \{\pi_1^*, \pi_2^*, \pi_3^*, q^*\}$ and the relative efficiencies of the optimal designs with equal group sizes for various τ_r, and $f_i, i = 1, 2$ when $(\gamma_{11}, \gamma_{21}, \gamma_{12}, \gamma_{22}) = (2.5, 1.5, 1, 0.5)$, $w_1 = 0.3$, $w_2 = 0.5$ and $\kappa = 0.5$.

τ_1	τ_2	f_1	f_2	f_3	Cost function 1					Cost function 2				
					π_1^*	π_2^*	π_3^*	q^*	RE	π_1^*	π_2^*	π_3^*	q^*	RE
1/3	1/3	1	1	1	0.38	0.29	0.33	2	0.99	0.38	0.29	0.33	2	0.99
1/3	1/3	1	1	100	0.37	0.30	0.33	8	0.99	0.37	0.30	0.33	12	0.99
1/3	1/3	1	100	100	0.37	0.30	0.33	12	0.99	0.37	0.30	0.33	12	0.99
1/3	1/3	50	50	50	0.37	0.30	0.33	11	0.99	0.37	0.30	0.33	12	0.99
1/3	1/3	50	100	100	0.37	0.30	0.33	12	0.99	0.37	0.30	0.33	12	0.99
1/3	1/3	100	100	100	0.37	0.30	0.33	12	0.99	0.37	0.30	0.33	12	0.99
3	1/3	1	1	1	0.38	0.29	0.33	12	0.99	0.38	0.29	0.33	12	0.99
3	1/3	1	1	100	0.38	0.29	0.33	12	0.99	0.38	0.29	0.33	12	0.99
3	1/3	1	100	100	0.38	0.29	0.33	12	0.99	0.38	0.29	0.33	12	0.99
3	1/3	50	50	50	0.38	0.29	0.33	12	0.99	0.38	0.29	0.33	12	0.99
3	1/3	50	100	100	0.38	0.29	0.33	12	0.99	0.38	0.29	0.33	12	0.99
3	1/3	100	100	100	0.38	0.29	0.33	12	0.99	0.38	0.29	0.33	12	0.99
3	3	1	1	1	0.38	0.28	0.34	12	0.99	0.38	0.28	0.34	12	0.99
3	3	1	1	100	0.38	0.28	0.34	12	0.99	0.38	0.28	0.34	12	0.99
3	3	1	100	100	0.38	0.28	0.34	12	0.99	0.38	0.28	0.34	12	0.99
3	3	50	50	50	0.38	0.28	0.34	12	0.99	0.38	0.28	0.34	12	0.99
3	3	50	100	100	0.38	0.28	0.34	12	0.99	0.38	0.28	0.34	12	0.99
3	3	100	100	100	0.38	0.28	0.34	12	0.99	0.38	0.28	0.34	12	0.99

Table S15 D_{s_2}-optimal designs $\zeta^* = \{\pi_1^*, \pi_2^*, \pi_3^*, q^*\}$ and the relative efficiencies of the optimal designs with equal group sizes for various τ_r, and $f_i, i = 1, 2$ when $(\gamma_{11}, \gamma_{21}, \gamma_{12}, \gamma_{22}) = (2.5, 1.5, 1, 0.5)$, $w_1 = 0.3$, $w_2 = 0.5$ and $\kappa = 0.5$.

τ_1	τ_2	f_1	f_2	f_3	Cost function 1					Cost function 2				
					π_1^*	π_2^*	π_3^*	q^*	RE	π_1^*	π_2^*	π_3^*	q^*	RE
1/3	1/3	1	1	1	0.59	0.40	0.01	2	0.65	0.59	0.40	0.01	2	0.65
1/3	1/3	1	1	100	0.58	0.41	0.01	8	0.66	0.57	0.42	0.01	12	0.66
1/3	1/3	1	100	100	0.57	0.42	0.01	12	0.66	0.57	0.42	0.01	12	0.66
1/3	1/3	50	50	50	0.57	0.42	0.01	10	0.66	0.57	0.42	0.01	12	0.66
1/3	1/3	50	100	100	0.57	0.42	0.01	12	0.66	0.57	0.42	0.01	12	0.66
1/3	1/3	100	100	100	0.57	0.42	0.01	12	0.66	0.57	0.42	0.01	12	0.66
3	1/3	1	1	1	0.59	0.40	0.01	11	0.66	0.58	0.41	0.01	12	0.67
3	1/3	1	1	100	0.58	0.41	0.01	12	0.67	0.58	0.41	0.01	12	0.67
3	1/3	1	100	100	0.58	0.41	0.01	12	0.67	0.58	0.41	0.01	12	0.67
3	1/3	50	50	50	0.58	0.41	0.01	12	0.67	0.58	0.41	0.01	12	0.67
3	1/3	50	100	100	0.58	0.41	0.01	12	0.67	0.58	0.41	0.01	12	0.67
3	1/3	100	100	100	0.58	0.41	0.01	12	0.67	0.58	0.41	0.01	12	0.67
3	3	1	1	1	0.59	0.40	0.01	12	0.68	0.59	0.40	0.01	12	0.68
3	3	1	1	100	0.59	0.40	0.01	12	0.68	0.59	0.40	0.01	12	0.68
3	3	1	100	100	0.59	0.40	0.01	12	0.68	0.59	0.40	0.01	12	0.68
3	3	50	50	50	0.59	0.40	0.01	12	0.68	0.59	0.40	0.01	12	0.68
3	3	50	100	100	0.59	0.40	0.01	12	0.68	0.59	0.40	0.01	12	0.68
3	3	100	100	100	0.59	0.40	0.01	12	0.68	0.59	0.40	0.01	12	0.68

Table S16 D_{s_3}-optimal designs $\zeta^* = \{\pi_1^*, \pi_2^*, \pi_3^*, q^*\}$ and the relative efficiencies of the optimal designs with equal group sizes for various τ_r, and $f_i, i = 1, 2$ when $(\gamma_{11}, \gamma_{21}, \gamma_{12}, \gamma_{22}) = (2.5, 1.5, 1, 0.5)$, $w_1 = 0.3$,$w_2 = 0.5$ and $\kappa = 0.5$.

τ_1	τ_2	f_1	f_2	f_3	Cost function 1					Cost function 2				
					π_1^*	π_2^*	π_3^*	q^*	RE	π_1^*	π_2^*	π_3^*	q^*	RE
1/3	1/3	1	1	1	0.55	0.01	0.44	2	0.66	0.55	0.01	0.44	2	0.66
1/3	1/3	1	1	100	0.55	0.01	0.44	10	0.67	0.55	0.01	0.44	12	0.67
1/3	1/3	1	100	100	0.55	0.01	0.44	12	0.67	0.55	0.01	0.44	12	0.67
1/3	1/3	50	50	50	0.55	0.01	0.44	12	0.67	0.55	0.01	0.44	12	0.67
1/3	1/3	50	100	100	0.55	0.01	0.44	12	0.67	0.55	0.01	0.44	12	0.67
1/3	1/3	100	100	100	0.55	0.01	0.44	12	0.67	0.55	0.01	0.44	12	0.67
3	1/3	1	1	1	0.56	0.01	0.43	12	0.66	0.56	0.01	0.43	12	0.66
3	1/3	1	1	100	0.56	0.01	0.43	12	0.66	0.56	0.01	0.43	12	0.66
3	1/3	1	100	100	0.56	0.01	0.43	12	0.66	0.56	0.01	0.43	12	0.66
3	1/3	50	50	50	0.56	0.01	0.43	12	0.66	0.56	0.01	0.43	12	0.66
3	1/3	50	100	100	0.56	0.01	0.43	12	0.66	0.56	0.01	0.43	12	0.66
3	1/3	100	100	100	0.56	0.01	0.43	12	0.66	0.56	0.01	0.43	12	0.66
3	3	1	1	1	0.56	0.01	0.43	12	0.66	0.56	0.01	0.43	12	0.66
3	3	1	1	100	0.56	0.01	0.43	12	0.66	0.56	0.01	0.43	12	0.66
3	3	1	100	100	0.56	0.01	0.43	12	0.66	0.56	0.01	0.43	12	0.66
3	3	50	50	50	0.56	0.01	0.43	12	0.66	0.56	0.01	0.43	12	0.66
3	3	50	100	100	0.56	0.01	0.43	12	0.66	0.56	0.01	0.43	12	0.66
3	3	100	100	100	0.56	0.01	0.43	12	0.66	0.56	0.01	0.43	12	0.66

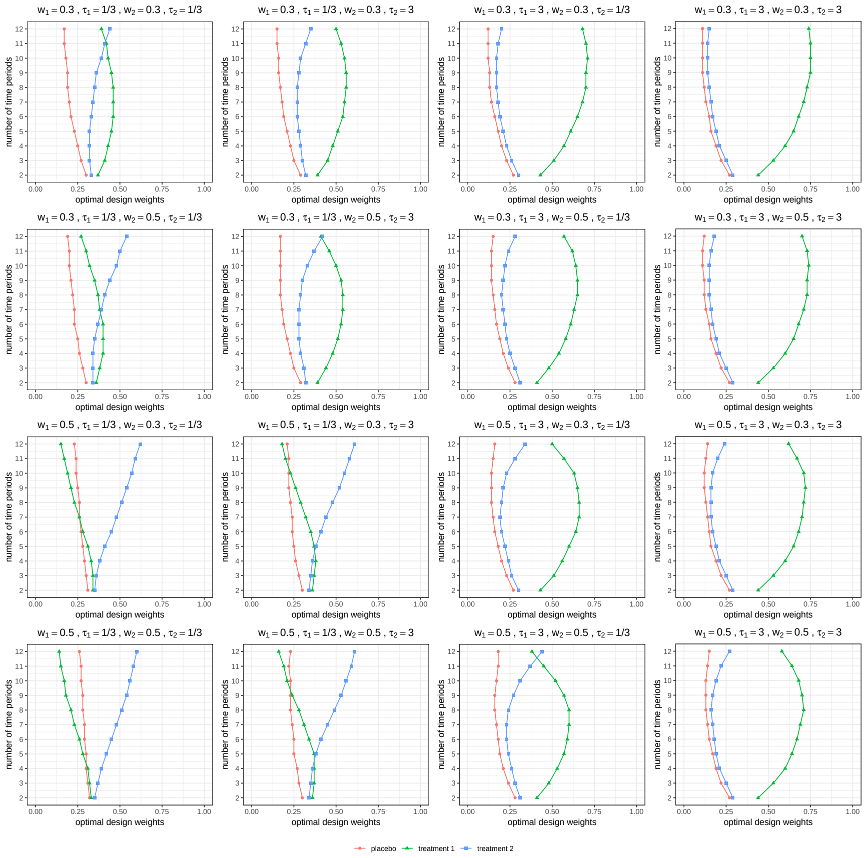

Fig. S5 Weights of D-optimal design for estimating all parameters in the model for different number of time periods q, proportions of event occurrence $w_r, r = 1, 2$, and shape parameters $\tau_r, r = 1, 2$ when $(\gamma_{11}, \gamma_{21}, \gamma_{12}, \gamma_{22}) = (2.5, 1.5, 1, 0.5)$ and $\kappa = 0.5$.

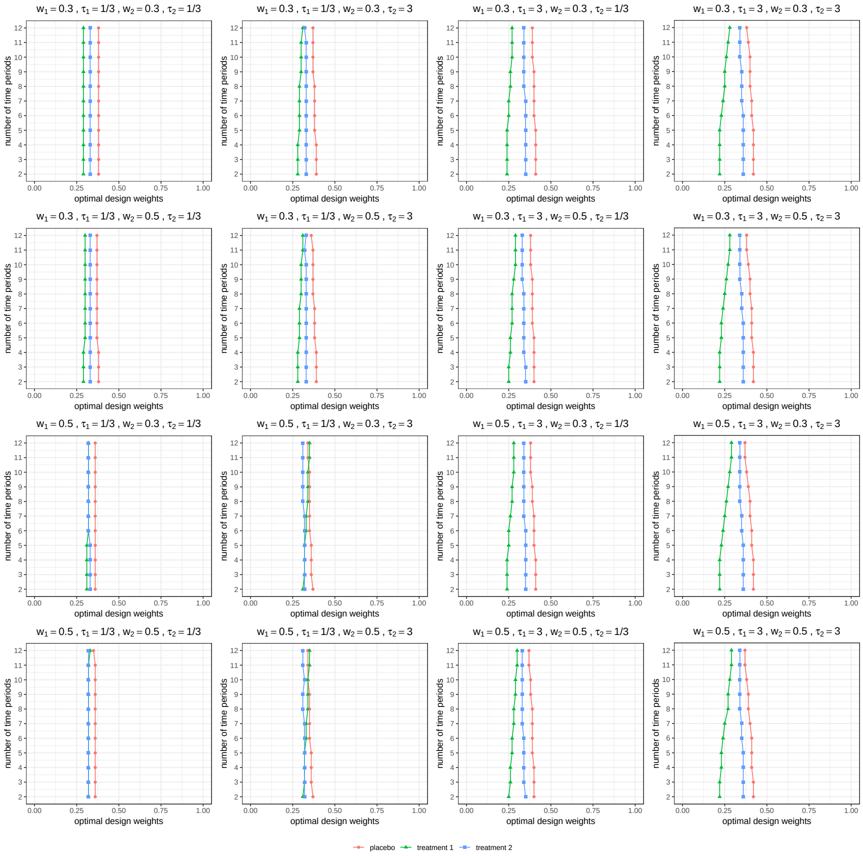

Fig. S6 Weights of D_{s_1}-optimal design for estimating all treatment effects for different number of time periods q, proportions of event occurrence $w_r, r = 1, 2$, and shape parameters $\tau_r, r = 1, 2$ when $(\gamma_{11}, \gamma_{21}, \gamma_{12}, \gamma_{22}) = (2.5, 1.5, 1, 0.5)$ and $\kappa = 0.5$.

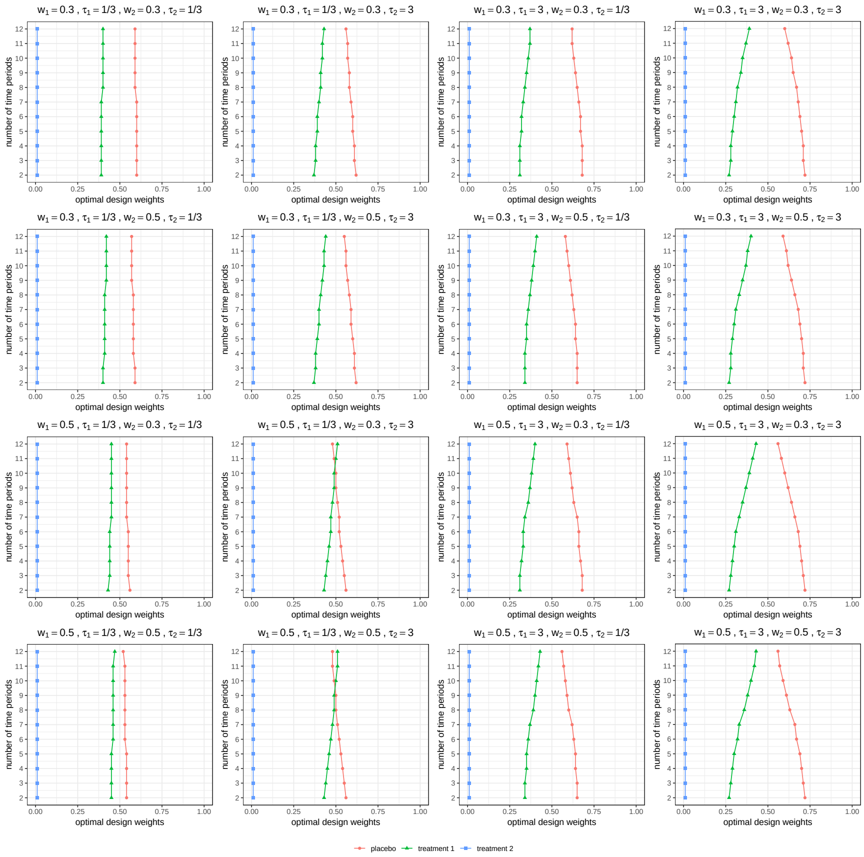

Fig. S7 Weights of D_{s_2}-optimal designs for estimating the effects of treatment 1 for different number of time periods q, proportions of event occurrence $w_r, r = 1, 2$, and shape parameters $\tau_r, r = 1, 2$ when $(\gamma_{11}, \gamma_{21}, \gamma_{12}, \gamma_{22}) = (2.5, 1.5, 1, 0.5)$ and $\kappa = 0.5$.

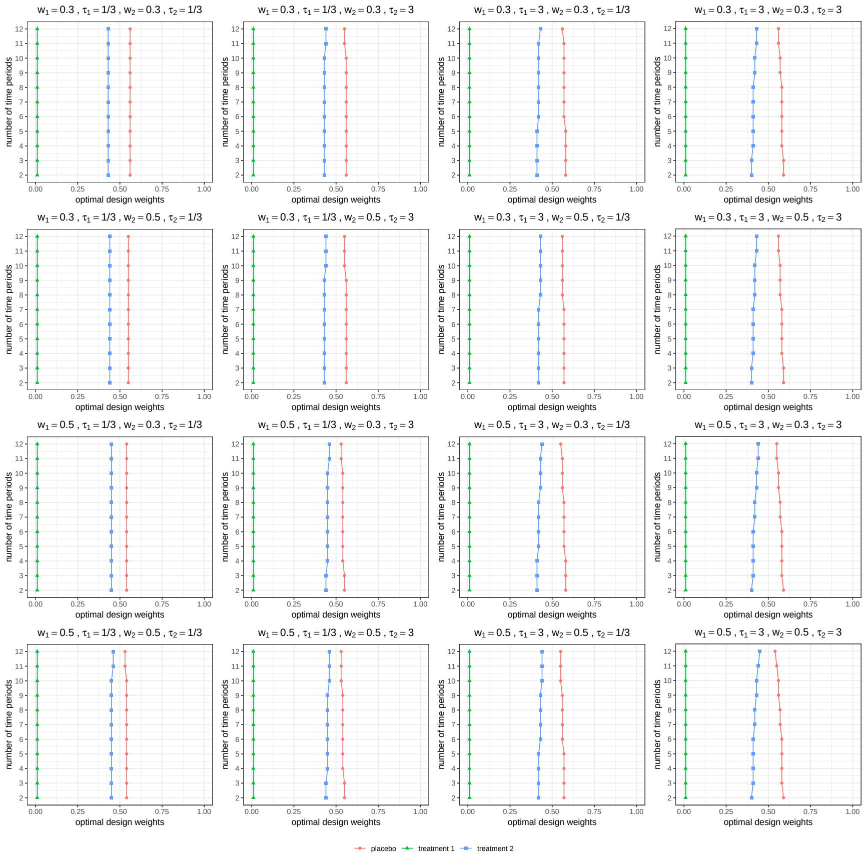

Fig. S8 Weights of D_{s_3}-optimal design for estimating the effects of treatment 2 for different number of time periods q, proportions of event occurrence $w_r, r = 1, 2$, and shape parameters $\tau_r, r = 1, 2$ when $(\gamma_{11}, \gamma_{21}, \gamma_{12}, \gamma_{22}) = (2.5, 1.5, 1, 0.5)$ and $\kappa = 0.5$.
